# Persistent sexually dimorphic effects of adolescent THC exposure on hippocampal synaptic plasticity and episodic memory in rodents

**DOI:** 10.1016/j.nbd.2021.105565

**Published:** 2021-11-25

**Authors:** Aliza A. Le, Julian Quintanilla, Mohammad Amani, Daniele Piomelli, Gary Lynch, Christine M. Gall

**Affiliations:** a Departments of Anatomy & Neurobiology, University of California, Irvine, CA 92697, United States of America; b Departments of Psychiatry & Human Behavior, University of California, Irvine, CA 92868, United States of America; c Departments of Neurobiology & Behavior, University of California, Irvine, CA 92697, United States of America

**Keywords:** THC, hippocampus, CA1, Lateral perforant path, Cannabinoid, Spatial learning, Long-term potentiation, Frequency facilitation, Estrogen, Sex differences

## Abstract

There is evidence that cannabis use during adolescence leads to memory and cognitive problems in young adulthood but little is known about effects of early life cannabis exposure on synaptic operations that are critical for encoding and organizing information. We report here that a 14-day course of daily Δ^9^-tetrahydrocannabinol treatments administered to adolescent rats and mice (aTHC) leads to profound but selective deficits in synaptic plasticity in two axonal systems in female, and to lesser extent male, hippocampus as assessed in adulthood. Adolescent-THC exposure did not alter basic synaptic transmission (input/output curves) and had only modest effects on frequency facilitation. Nevertheless, aTHC severely impaired the endocannabinoid-dependent long-term potentiation in the lateral perforant path in females of both species, and in male mice; this was reliably associated with impaired acquisition of a component of episodic memory that depends on lateral perforant path function. Potentiation in the Schaffer-commissural (S-C) projection to field CA1 was disrupted by aTHC treatment in females only and this was associated with both a deficit in estrogen effects on S-C synaptic responses and impairments to CA1-dependent spatial (object location) memory. In all the results demonstrate sexually dimorphic and projection system-specific effects of aTHC exposure that could underlie discrete effects of early life cannabinoid usage on adult cognitive function. Moreover they suggest that some of the enduring, sexually dimorphic effects of cannabis use reflect changes in synaptic estrogen action.

## 1. Introduction

Epidemiological studies have shown that cannabis use during adolescence increases the incidence of memory and cognitive problems later in life and experimental work has described analogous effects in animals (Ellgren et al., 2008; Schweinsburg et al., 2008; Rubino and Parolaro, 2011; Meier et al., 2012; Renard et al., 2014; Blest-Hopley et al., 2020). Cannabis-associated alterations that could contribute to psychological and cognitive disturbances include changes in cortical morphology (Wilson et al., 2000; Mata et al., 2010; Ashtari et al., 2011; Tomasiewicz et al., 2012), ascending monoamine systems (Bambico et al., 2010; Renard et al., 2017), network activity (Ruiz et al., 2020), and epigenetic adjustments (Tomasiewicz et al., 2012; Szutorisz and Hurd, 2016). Surprisingly, little is known about enduring effects of cannabis exposure on synaptic function in forebrain regions that are critical for memory formation. In rat, early life Δ^9^-tetrahydrocannabinol (THC) treatment has been shown to depress GABAergic transmission and endocannabinoid-dependent long-term depression (LTD) in frontal cortex without affecting long-term potentiation (LTP) in the same fields (Zamberletti et al., 2014; Rubino et al., 2015). Comparable physiological results are not available for hippocampus although adolescent THC (aTHC) exposure reduces spine densities in the dentate gyrus (Wilson et al., 2000; Rubino et al., 2009). Whether such disturbances are accompanied by abnormalities in functional synaptic plasticity is not known.

The above question is important for understanding effects of early cannabis use on everyday life. The hippocampus is critical for acquisition of spatial information (Tsien et al., 1996; Seese et al., 2012; Seese et al., 2014) and episodic sequences (Squire, 1992; Westmacott et al., 2001; Eichenbaum, 2014; Dede et al., 2016). Episodic memory organizes the flow of daily experiences into narratives that include the identities, spatial relationships, and temporal order of elements encountered (‘what’, ‘where’, and ‘when’ information) (Tulving, 1983; Ergorul and Eichenbaum, 2004). Moreover, episodic memories influence higher order cognitive processes including inferential and prospective thinking (Crane and Goddard, 2008) and repeated cannabis use during adolescence is associated with lasting impairments to episodic memory (Rubino et al., 2009; Crane et al., 2013a; Riba et al., 2015). Accordingly, the present studies tested if, in rodents, a 14-day aTHC treatment regimen has enduring effects on synaptic operations at two nodes in hippocampal circuitry and the related encoding of episodic and spatial memory.

Behavioral effects of adolescent cannabinoid exposure differ between the sexes in humans and rodents, with more severe disruptions typically reported for females (Cohn et al., 1972; Rubino et al., 2009; Fattore and Fratta, 2010; Lovelace et al., 2015; Rubino et al., 2015; Silva et al., 2016; Calakos et al., 2017; Borsoi et al., 2019; Ruiz et al., 2020; Bara et al., 2021). Factors responsible for these sex differences are not known although interactions between cannabinoid exposure and male-female variations in brain subsystems are suggested to contribute (Rubino et al., 2008; Rubino and Parolaro, 2011; Silva et al., 2016; Djurisic et al., 2019). This argument is of particular interest regarding hippocampus because field CA1 LTP is strongly sexually dimorphic and depends upon membrane estrogen receptor signaling in females only (Vierk et al., 2012; Wang et al., 2018c). The requirement for estrogen signaling elevates the threshold for CA1 LTP in non-proestrus females relative to males, a finding that aligns with sex differences in CA1-dependent memory formation (Andreano and Cahill, 2009; Asperholm et al., 2019; Gall et al., 2021). Assuming that enduring consequences of aTHC on memory are shaped by sex differences in synaptic plasticity, such treatments may differentially affect female and male LTP in hippocampus. The present studies tested this possibility for the CA3 to CA1 Schaffer-commissural (S-C) projections as well as for the lateral perforant path (LPP) afferents from lateral entorhinal cortex to the dentate gyrus outer molecular layer. Potentiation within the S-C system is modulated by endocannabinoids (Wang et al., 2016b; Wang et al., 2018a; Wang et al., 2018b), in part due to effects on GABAergic systems (Carlson et al., 2002; Chevaleyre and Castillo, 2004; Huang and Woolley, 2012), but as determined in studies of using antagonists and receptor knockout mice, enduring potentiation in this system is not dependent upon endocannabinoid signaling (Bohme et al., 2000; Monory et al., 2015; Wang et al., 2016a; Wang et al., 2016b; Wang et al., 2018b). In contrast, in rats and mice the expression of LPP LTP requires endocannabinoid and cannabinoid receptor 1 (CB_1_R) signaling (Wang et al., 2016b; Wang et al., 2018b; Wang et al., 2018c). Moreover, the present studies evaluated effects in both rats and mice to align results with prior findings on cannabinoid metabolism and action in these animals, and to avoid undue focus on effects that may be species specific. The results demonstrate that aTHC exposure causes striking but distinct and sexually dimorphic LTP impairments in the two axonal systems in both rats and mice, and these are predictive for equally dramatic deficits in memory formation.

## 2. Materials and methods

Studies used male and female Long-Evans rats and C57BL/6 N mice (Envigo, Placentia CA) obtained from vendors separated by sex and at least one week prior to experimental use. Animals were group-housed (4 rats/cage; 5 mice/cage) with water and food ad libitum. Procedures were in accordance with the NIH Guide for the Care and Use of Laboratory Animals and institutionally approved protocols.

### THC Administration.

Animals received intraperitoneal injections of vehicle (5% Tween in 0.9% saline, 2 mL/kg) or THC (5 mg/kg; Cayman Chemicals) daily from postnatal day (P) 30 to P43 and were allowed 21 days of THC washout before initiating electrophysiological and behavioral studies. As assessed in adult rodents, this dose yields comparable blood and brain THC levels in males and females (Ruiz et al., 2020; Torrens et al., 2020) and, as assessed in adult male mice, yields maximal plasma THC levels that are similar to those achieved in persons who smoked a single cannabis cigarette containing 34 mg of Δ^9^-THC (Huestis and Cone, 2004; Torrens et al., 2020). Moreover this dose matches the proposed ‘Standard THC Unit’ to be used in research reports on cannabis products and methods administration (Freeman and Lorenzetti, 2020).

### Estrous Staging.

All females were evaluated for estrous state using vaginal smears (Caligioni, 2009; McLean et al., 2012; Cora et al., 2015). For electrophysiology, smears were collected at sacrifice. As rat electrophysiological measures did not differ between animals within and outside proestrus for LTP in the S-C system (unpaired *t-test*: Vehicle *P* = 0.93, THC: *P* = 0.19) or the LPP (Vehicle *P* = 0.99, THC *P* = 0.16), results from females at the different cycle stages were pooled for statistical analyses and illustrations within the text. Mice used for electrophysiology were all outside proestrus. Results from the same rats and mice are shown plotted by estrous stage in Supplementary Figs. 2 and 3. For behavioral studies, estrous stage was assessed daily for at least 3 days before experimental use to assure cycling and that all animals were trained outside proestrus (i.e., in the lower circulating estrogen states of estrus or diestrus (Hojo and Kawato, 2018).

### 2.1. Electrophysiology

Hippocampal slice preparation and field recordings followed published procedures (Trieu et al., 2015; Wang et al., 2016b) with transverse sectioning (360 μm) using a McIllwain tissue chopper and collection into chilled artificial cerebrospinal fluid (aCSF) containing in mM: 124 NaCl, 3 KCl, 1.25 KH_2_PO_4_, 1.5 MgSO_4_, 26 NaHCO_3_, 2.5 CaCl_2_, and 10 dextrose. Slices were promptly transferred to an interface recording chamber maintained at 31 ± 1 °C with constant 60–70 mL/h perfusion of oxygenated aCSF; experiments were initiated 1.5–2 h later.

#### CA1 S-C System.

To monitor S-C responses in field CA1, bipolar stimulating (twisted nichrome) and glass pipette (2 M NaCl) recording electrodes were placed in CA1a and CA1b stratum radiatum, respectively (Wang et al., 2018c). Stimulation strength was adjusted to elicit field excitatory postsynaptic potential (fEPSP) amplitudes at ~50% of the maximum population-spike free response; initial response slopes were collected using NacGather 2.0 (Theta burst Corp). For studies of synaptic potentiation, baseline responses to 0.05 Hz stimulation were recorded for 20 min before applying theta-burst stimulation (TBS: bursts of 4 pulses at 100 Hz, 200 ms between bursts); rats received a single train of 10 bursts (Larson et al., 1986), whereas mice received 5 bursts (Lauterborn et al., 2007), train lengths previously shown to be nearthreshold for the induction of LTP in the two species. Following TBS, responses to 0.05 Hz stimulation were collected for 60–80 min. To assess estradiol (E2) effects on CA1 responses, E2 (β-estradiol, Tocris; 1 nM) was infused alone or with estrogen receptor α (ERα) antagonist methyl-piperidino-pyrazole (MPP, Tocris; 3 μM) (Wang et al., 2016a).

#### LPP.

Stimulating and recording electrodes were placed in the outer third of the dentate gyrus molecular layer (internal blade). Paired-pulse stimulation confirmed electrode placement as this distinguishes responses of the lateral and adjacent medial perforant paths (Wang et al., 2018c). To induce LPP potentiation, 1 s of 100 Hz, high-frequency stimulation (HFS) was applied with pulse duration doubled and intensity increased x1.5 relative to baseline stimulation; 0.05 Hz stimulation resumed thereafter.

For text illustrations and statistical analyses, mean responses were normalized to the first pulse response for frequency facilitation analysis and to the first within-burst response for complex spike analysis to eliminate the between-run variability in absolute response measures that can be attributed to properties and placements of the stimulation and recording electrodes. Plots of raw measures are presented in supplemental illustrations as indicated in the Results. The level of potentiation for both S-C and LPP systems was determined by averaging, for individual slices, fEPSP slopes for the first 2 min post-induction for short-term potentiation and for 55–60 min post-induction for LTP, normalized to the within-slice mean response during the last 5 min of baseline recordings. The within-slice potentiation measures were averaged for a given experimental group and presented as group mean ± SEM values in text illustrations.

### 2.2. Behavior

#### General procedures for behavioral studies.

Just before training/testing sessions for the Serial ‘What’ and 2-Odor discrimination tasks, 100 μL of a single odorant (Table 1) was pipetted onto a filter paper within a glass cup (Mouse cups: 5.25 cm diam x 5 cm high; Rat cups: 6.5 cm diam x 6 cm high) with a perforated lid. The odors chosen for these behaviors have been verified to have equal salience to mice (Amani et al., 2021) and rats (Quintanilla et al., 2021). Behavioral tests were counter-balanced for location of odors and treatments. Testing arenas and odor cups/objects were cleaned with 70% ethanol between subjects. For all behavioral sessions, animal movements were recorded using an overhead camera. For Serial ‘What’ and 2-Odor discrimination tasks, a rodent was considered to be exploring an odor when its nose was directly above the cup and oriented toward the odor holes; instances when the animal sat on top of the cups were not counted and animals that attended to any cup for less than 1 s were excluded from analysis. For the object location memory task, time exploring an object was recorded when the animal faced and sniffed toward the object; time near but looking away from the object was not scored. Text illustrations show group mean ± SEM values; behavioral measures for individual animals, including estrous staging for females, are presented in Supplemental materials.

#### *Serial ‘What’ Task* Wang et al., 2018a;Cox et al., 2019).

Animals were habituated to an empty testing chamber (Plexiglas, 40x40x40 cm for rats, 30x25x21.5 cm for mice) in 2–3 daily 5-min sessions. On the test day, rats were allowed to explore the chamber containing two unscented cups (in opposing corners) for 2 min. Cups were removed and replaced 2 min later with two cups scented with odor A and the rat was allowed to explore for 2 min. This was followed by sequential exposure to three more odor pairs (B:B; C:C; D:D) with 2-min exposure and 5-min spacing. After a final break, rats were exposed to previously sampled odor A and novel odor E for 3 min. The time spent exploring A vs E was determined from videotapes by observers blind to group, and the percent times sampling the novel and familiar odors were compared. The task was similar for mice but entailed training-exposure to three odor pairs for 3 min each, with 3-min intervals between pairs, and a 3-min test session with previous-odor A and novel-odor D.

#### 2-Odor Discrimination.

Rats habituated to the arena and unscented cups were exposed to identical odor pair F:F for 2 min and, after a 26-min delay, were presented with test odors F(familiar):G(novel); the times spent exploring these odors over the next 3 min were analyzed (Wang et al., 2018a; Cox et al., 2019). Procedures were similar for mice excepting 3-min initial odor-pair presentation and exposure to the test odor pair (E:F) after a 15-min delay.

#### *Object Location Memory Task* (Seese et al., 2014; Wang et al., 2018c).

Rats were habituated to the empty arena (with spatial cues on walls) for 5-min on 2 consecutive days. The next day, rats were placed in the arena containing two identical objects (glass vases) placed near adjacent corners and allowed to explore for 5 min timed from first object interaction. For testing 24 h later, the animal was placed in the arena with one object moved toward the center and allowed to explore for 5 min. For mice, test objects were clear glass funnels, and the initial object exposure period approximated threshold for learning by males (5-min) and non-proestrus females (10-min) (Seese et al., 2014; Wang et al., 2018c). For all groups, the discrimination index (DI) was calculated as follows: 100*(time_displaced object_–time_stationary object_)/(time_total_) for the first 3 min of the test period.

#### Statistical analyses and data presentation.

Results plotted are group mean ± SEM values unless otherwise stated. For all electrophysiological studies, the group N is the number of slices studied from a minimum of 3 animals. Significance was determined using the one or two-tailed Student’s *t-test*, repeated-measures (RM) ANOVA (Interaction), one-way ANOVA with Bonferroni post hoc test for paired comparisons, or linear regression, all using GraphPad Prism 6.0 software. For statistical comparisons, *p* < 0.05 was considered significant.

## 3. Results

**Fig. 1.**
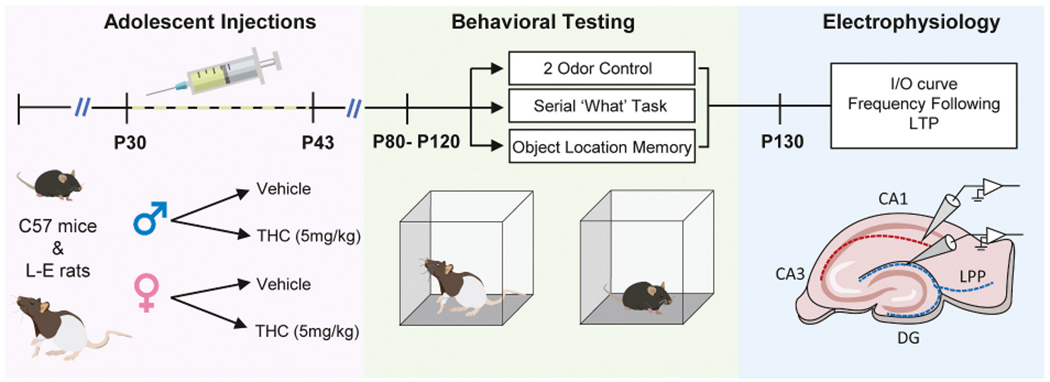
Experimental design and timeline. C57BL6 mice and Long-Evans (L-E) rats of both sexes received daily injections with vehicle or 5 mg/kg THC from postnatal day (P) 30-P43. All groups were allowed at minimum a 3-week washout period before experimental use. After this time, behavior was assessed in 3 tasks (2 odor control, Serial ‘What’ Task, Object Location Memory), one week apart. The specific task order was randomized by cohort (each cohort included equal numbers of vehicle and aTHC-treated animals). Upon completion of behavioral tasks, animals were used for electrophysiological analyses targeting the S—C projection to CA1 apical dendrites and lateral perforant path (LPP) innervation of the dentate gyrus (DG).

Studies used field recordings from acute hippocampal slices to assess in both rats and mice effects of adolescent THC treatment (5 mg/kg daily from P30–43) (aTHC) on increasingly complex aspects of synaptic communication within the adult hippocampus: input/output relationships, frequency facilitation to basic rhythms that occur during behavior, responses to TBS, and synaptic potentiation. Responses were evaluated for S-C innervation of CA1b stratum radiatum and LPP innervation of the dentate gyrus outer molecular layer. We also evaluated effects of aTHC on learning in paradigms that rely on the function of these particular projection systems. The timeline for animal procedures is illustrated in Fig. 1.

### 3.1. Input-output curves

**Fig. 2.**
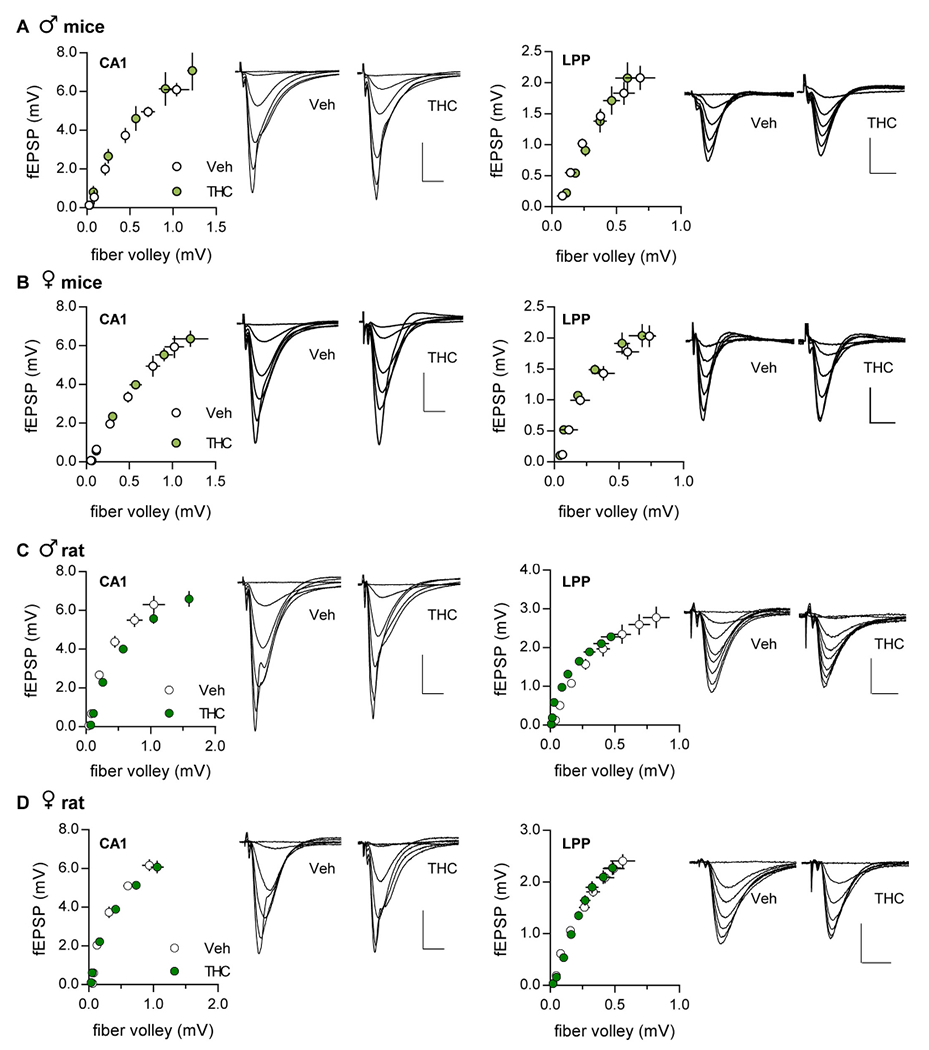
Input/output (I/O) curves for the S—C and LPP systems were not influenced by adolescent THC treatment. I/O curves were comparable between vehicle (Veh) and aTHC-treated animals for both the S—C CA1 and lateral perforant path (LPP) systems in (A) male mice (CA1: *p* = 0.38, F_(1,62)_ = 0.80; LPP: *p* = 0.05, F_(1,74)_ = 3.9, *N* = 5–6), (B) female mice (CA1: *p* = 0.39, F_(1,73)_ = 0.75; LPP: *p* = 0.56, F_(1,56)_ = 0.35, *N* = 5–7), (C) male rats (CA1: *p* = 0.15, F_(1,8)_ = 2.56; LPP: *p* = 0.10, F_(1,14)_ = 3.02, *N* = 7–8), and (D) female rats (CA1: *p* = 0.51, F_(1,8)_ = 0.47; LPP: *p* = 0.18, F_(1,14)_ = 1.20, *N* = 9–13). Linear regression was used to evaluate significance; mean ± SEM values shown. Scale bars for CA1 traces were 2 mV, 5 ms and for LPP traces were 1 mV, 5 ms. N values denote slices per group.

The relationship between the numbers of axons stimulated to the size of the postsynaptic response was assessed by measuring the amplitudes of fiber volleys and fEPSPs evoked by ascending stimulation currents. There were no significant effects of aTHC on these measures for either S-C or LPP systems (Fig. 2).

### 3.2. Frequency facilitation

**Fig. 3.**
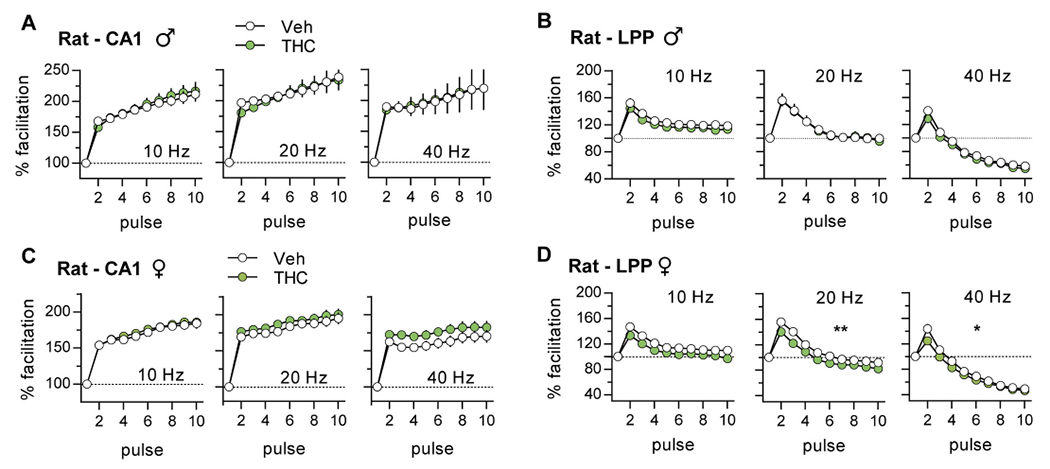
Adolescent THC treatment had little effect on frequency facilitation (FF) in rat S—C (CA1) and LPP systems. (A,B) In slices from adult male rats given vehicle (Veh) or THC during adolescence, response profiles were comparable for S—C (A; 10 Hz: *p* = 0.87, F_(9,135)_ = 0.51; 20 Hz: *p* = 0.98, F_(9,135)_ = 0.29; 40 Hz: *p* = 0.99, F_(9,135)_ = 0.12. Veh *N* = 10, THC N = 7) and LPP (B; 10 Hz: *p* = 0.86, F_(9,117)_ = 0.52; 20 Hz: p = 0.99, F_(9,117)_ = 0.13; 40 Hz: *p* = 0.69, F_(9,117)_ = 0.72. Veh N = 7, THC *N* = 8) systems. (C,D) THC did not influence FF of the female S—C system (C; 10 Hz: p = 0.98, F_(9,234)_ = 0.29, *N* = 14/group; 20 Hz: *p* = 0.92, F_(9,225)_ = 0.42, Veh *N* = 11, THC *N* = 13; 40 Hz: *p* = 0.43, F_(9,234)_ = 1.01, N = 14/group) and only modestly depressed LPP FF with 20 and 40 Hz stimulation (D; 10 Hz: *p* = 0.06, F_(9,270)_ = 1.88; 20 Hz: **p = 0.009, F_(9,270)_ = 2.53; 40 Hz: *p = 0.01, F_(9,270)_ = 2.39, *N* = 16/group). N values are slices per group; mean ± SEM values shown. Statistics used RM-ANOVA (Interaction). Raw values are reported in Suppl. Figs. 1,2.

**Fig. 4.**
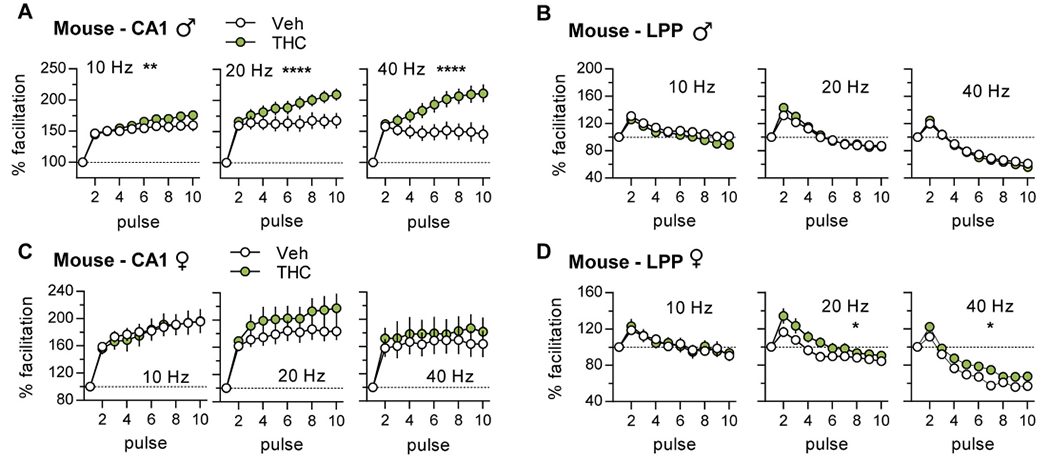
Adolescent THC (aTHC) treatment enhanced frequency facilitation (FF) of S—C (CA1) projections in male mice. Ten pulse trains of 10, 20, or 40 Hz stimulation were applied to S—C or LPP systems and initial fEPSP slopes were normalized to the first response. (A) For males, S—C FF was greater at all frequencies for aTHC vs Vehicle (Veh) groups (10 Hz: **p = 0.002, F_(9,108)_ = 3.20; 20 Hz: ****p < 0.0001, F_(9,108)_ = 5.76; 40 Hz: ****p < 0.0001, F_(9,108)_ = 9.59, N = 7/group). (B) For male LPP, FF was comparable between Veh- and aTHC-treatment groups (10 Hz: *p* = 0.13, F_(9,117)_ = 1.56; 20 Hz: *p* = 0.08, F_(9,108)_ = 1.76; 40 Hz: *p* = 0.94, F(9,117) = 0.39, Veh N = 8, THC *N* = 6–7). (C,D) Among females, aTHC did not alter S—C FF (C; 10 Hz: p = 0.99, F_(9,108)_ = 0.22, N = 14/group; 20 Hz: *p* = 0.31, F_(9,108)_ = 1.20, Veh N = 11, THC N = 13; 40 Hz: *p* = 0.98, F_(9,108)_ = 0.26, N = 7/group), but enhanced LPP FF with 20 and 40 Hz stimulation (D; 10 Hz: *p* = 0.74, F_(9,90)_ = 0.67; 20 Hz: *p = 0.02, F_(9,90)_ = 2.3; 40 Hz: *p = 0.049, F_(9,90)_ = 1.99, N = 6/group). N denotes slices per group; mean ± SEM values shown. Statistics used RM-ANOVA (Interaction). Raw values for these datasets are presented in Suppl. Figs. 1,2.

Repetitive synaptic activation typically results in enhanced transmitter release and an increase in the initial slope of postsynaptic potentials. Such frequency facilitation (FF) is evident throughout the nervous system (Job and Lundberg, 1953; Liley and North, 1953; Andersen, 1960a; Andersen, 1960b; Lomo, 1971; Richards, 1972) and is thought to be a fundamental characteristic of secure interneuronal communication (Jackman and Regehr, 2017). We tested if aTHC influenced FF using a ten-pulse train of 10, 20, and 40 Hz stimulation. Figs. 3 and 4 show group mean values of fEPSP slopes in response to pulses 2 to 10 normalized to the first response; these results are summarized in Table 2. Plots of raw measures from the same groups, including female estrous stage breakdown, are presented in Supplemental Figs. 1 and 2.

#### Rats:

In slices from vehicle (Veh)-treated male and female rats, there was robust FF of S-C fEPSPs at each test frequency. For the LPP, FF was less pronounced than in CA1 and declined after the second pulse with responses falling below baseline at 40 Hz. The latter response profile is thought to be due to an exhaustion of the readily releasable vesicle pool with closely-spaced stimulation (Zucker and Regehr, 2002). Adolescent THC did not alter the FF profile in male CA1 as shown with measures normalized to the first pulse (Fig. 3A) or plotted as raw values (Suppl. Fig. 1A). For the LPP, the normalized FF profile was unaffected by aTHC (Fig. 3B); although the plot of raw measures reflects lower response sizes in the aTHC group but this was evident from the first response (Suppl. Fig. 1C). For females, aTHC had no effect on FF in CA1 but it modestly reduced response size in the LPP with 20 Hz (*p* = 0.009) and 40 Hz (*p* = 0.01) stimulation (Fig. 3C,D; Suppl. Fig. 2A,C).

#### Mice:

The FF curves for vehicle-treated mice followed the rat pattern for both S-C and LPP systems. However, in contrast to rats, aTHC *enhanced* S-C FF in male mice and increased responses to 20 Hz stimulation in females although the latter effect was not statistically significant (Fig. 4A,C; Suppl. Fig. 1B, 2B). For the LPP, aTHC had no effect on FF in males but modestly increased facilitation at 20 and 40 Hz (*p* < 0.05) in females (Fig 4B,D; Suppl. Figs. 1D, 2D).

### 3.3. Complex response profiles

**Fig. 5.**
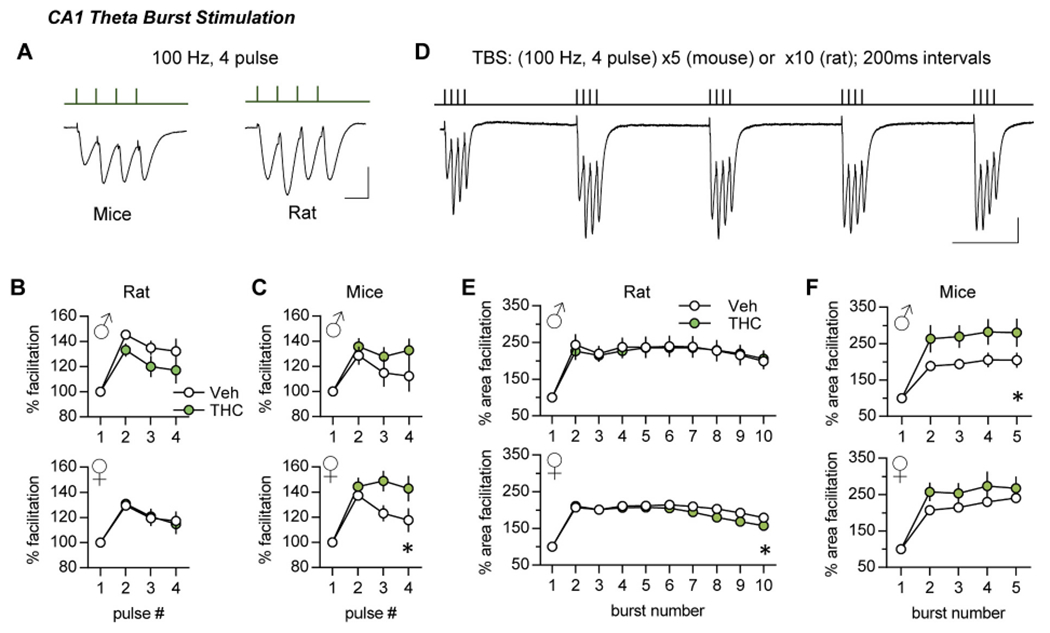
Adolescent THC treatment had modest effects on CA1 responses to burst stimulation and TBS trains. (A) Representative traces for a single burst response (100 Hz, 4 pulses). Scale bar: 1 mV, 10 ms. (B) The single burst response profile (fEPSP slopes, normalized to first pulse) did not differ between THC- and Veh-treated rats (Male: *p* = 0.44, F_(3,36)_ = 0.93, N = 7/group; Female: *p* = 0.96, F_(3,69)_ = 0.10, Veh N = 11, THC N = 14). (C) In mice, aTHC had no effect on the single burst response in males (*p* = 0.35, F(_3,30_) = 1.15, *n* = 6/group) but significantly enhanced responses to later pulses in the burst in females (*p = 0.02 vs Veh, F_(3,36)_ = 3.68, N = 7/group). (D) Representative trace from rat showing responses to the first 5 theta bursts (200 ms inter-burst). Scale bar: 1 mV, 100 ms. (E) In rat, THC did not influence within theta train facilitation in males (burst response area, normalized to first burst; p = 0.99 vs Veh, F_(9,108)_ = 0.23, N = 7/group) but caused a slight decline later in the theta train in females (*p = 0.03. F_(9,207)_ = 2.13, Veh N = 11, THC N = 14). (F) In mice, aTHC enhanced within theta train facilitation in males (*p = 0.02 vs Veh, F_(3,30)_ = 3.63, N = 6/group) without significant effect in females (*p* = 0.20, F_(3,36)_) = 1.62, N = 7/group). N denotes slices per group; mean ± SEM values shown. R-M ANOVA (Interaction) for all analyses. Raw measures are reported in Suppl. Fig. 3.

Hippocampal and neocortical pyramidal cells commonly fire in high frequency bursts during learning behavior (Ranck Jr., 1973; Fox and Ranck, 1975). Stimulation trains that mimic this pattern produce a composite postsynaptic response that reflects presynaptic frequency facilitation and spatio-temporal summation of EPSPs in target dendrites (Larson et al., 1986). In addition to contacting other pyramidal cells, the S-C projections contact GABAergic interneurons (Andersen et al., 1963), thereby forming di-synaptic feedforward inhibitory circuits (Andersen et al., 1963; Alger and Nicoll, 1982; Pouille and Scanziani, 2001; Jang et al., 2005). These arrangements result in partial shunting and thus GABAergic inhibition of later EPSPs in a burst response (Arai et al., 1995) (Fig. 5A). There were no measurable effects of aTHC on the complex response to a single high frequency burst of S-C stimulation in male or female rats, or male mice (Fig 5B,C). For female aTHC-treated mice, the later fEPSP slopes were larger than in vehicle cases suggesting attenuation of inhibition with cannabinoid treatment (Fig. 5C).

Single burst responses do not cause synaptic modifications, but multiple bursts separated by the period of the theta rhythm (~200 ms) are near optimal for inducing CA1 LTP (Larson et al., 1986) with the threshold to induction being somewhat lower in mouse than in rats. Thus, to provide near LTP-threshold stimulation we delivered to S-C projections a single train of 10 theta bursts for rat (Larson and Lynch, 1986) and 5 theta bursts for mouse (Lauterborn et al., 2007).

With TBS, the second burst response is greater than the first (Fig. 5D) largely due to suppression of feedforward shunting inhibition. The terminals of pertinent interneurons express GABA_B_ auto-receptors that, once activated by a first burst, depress subsequent GABA release with the maximal effect at 150–200 msec (Larson and Lynch, 1986; Davies et al., 1991). A second theta burst arriving after this interval thus produces depolarization that is sufficient to unblock voltage sensitive NMDARs (Larson and Lynch, 1988). Analysis of single and multiple theta burst responses in CA1 can accordingly detect influences on an increasing complex set of synaptic and local circuit operations. In rats aTHC did not influence within-train facilitation in males but slightly depressed this profile in females (Fig. 5E). In mice, aTHC increased within-train facilitation in males only (Fig 5F). See Supplementary Fig. 3A–F for raw measures.

In summary, for mice aTHC effects on the theta burst response profile in females, and theta train facilitation in males suggest that exposure to the cannabinoid reduced shunting inhibition (females) and disturbed metabotropic GABA_B_ autoinhibition and/or NMDAR activities (males), respectively.

### 3.4. Long-term potentiation (LTP)

#### S-C system:

**Fig. 6.**
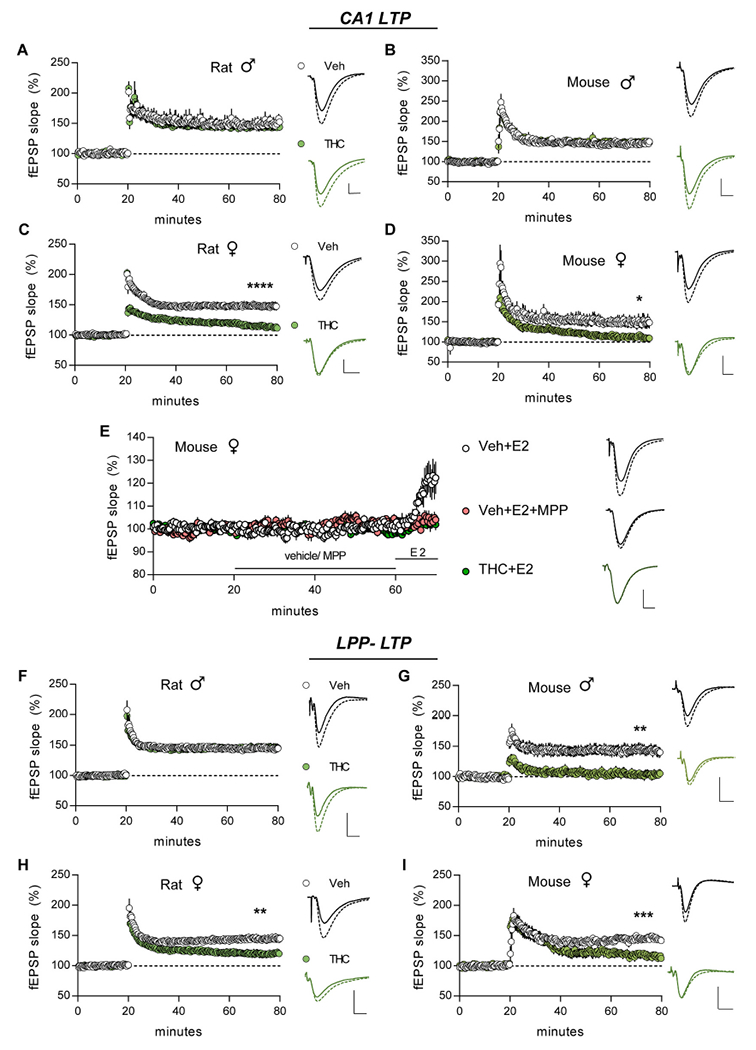
Adolescent THC impaired both S—C and LPP LTP with greatest effects in females. Potentiation was induced using a single train of near threshold TBS (5 bursts for mice; 10 bursts for rat) for S—C projections or 1 s of 100 Hz stimulation for the LPP. (A,B) In males, aTHC did not influence CA1-LTP in rats (A, *p* = 0.66, Veh N = 6 vs THC N = 7sliees/group) or mice (B, *p* = 0.70; N = 6/group). (C,D) In females, aTHC impaired S—C LTP in both rats (C, ****p < 0.0001, N = 13/group) and mice (D, *p = 0.01, N = 7/group). (E) In slices from Veh-treated female mice, infusion of estradiol (E2, 1 nM) increased the slopes of S—C fEPSPs and this effect was blocked in the presence of ERα antagonist MPP (3 μM) (One-way ANOVA: *p* = 0.004, F_(2,21)_ = 7.03; Bonferroni post-hoc: *p < 0.05 Veh + E2 vs E2 + MPP, N = 7/group). E2 failed to increase response size in slices from aTHC-treated female mice (post-hoc: **p < 0.01 Veh E2 vs THC + E2, N = 10). (F,G) In males, LPP LTP was unaffected by aTHC in rats (F, *p* = 0.77, N = 7/group) but was absent after aTHC treatment in mice (G, **p = 0.002; Veh N = 8 vs THC N = 7). (H,I) In female rats and mice, aTHC severely impaired LPP LTP (H, Rat: **p = 0.006, Veh *N* = 12 vs THC N = 14; I, Mouse: ***p = 0.0004, Veh N = 6 vs THC N = 7). For all panels, superimposed representative traces at right show mean baseline (min 18–20) (solid) and post-induction (min 78–80) (dashed) responses. N denotes slices per group. Mean ± SEM values shown. Scale bars: 1 mV, 5 ms. LTP statistics were analyzed with 2-tail unpaired *t-test*. Raw values are reported in Suppl. Fig. 3.

In slices from vehicle-treated animals, TBS applied to the S-C projections produced an immediate increase in the CA1 fEPSP slope; this short-term potentiation (STP) decayed over 5 min to plateau at ~50% above baseline (LTP). In male rats and mice, aTHC did not influence STP or LTP in this pathway (Fig. 6A,B). However, in female aTHC-treated rats, TBS failed to induce robust S-C STP (*p* = 0.005, aTHC vs Veh) and the modest potentiation decayed to near pre-induction values by 55–60 min post-TBS (Veh: +47.61 ± 6.16%, aTHC: +13.28 ± 2.84%; *p* < 0.0001) (Fig. 6C). Results for female mice nearly replicated those for female rats: S-C STP was reduced by aTHC and the subsequent potentiation decayed to near-baseline values over the next 60 min (*p* = 0.01, Veh vs aTHC) (Fig. 6D; Table 2). The marked deficits in CA1 STP and LTP indicate that in rat aTHC had enduring detrimental effects on triggering events that, in females, shift synapses into their stable potentiated state.

In males and females CA1 LTP is NMDAR-dependent (Larson and Lynch, 1988; Wang et al., 2016b) whereas in females only, LTP also depends upon locally produced estrogen (Vierk et al., 2012; Wang et al., 2018c) and signaling through membrane-associated ERα (Wang et al., 2018c). In adult females, treatment with 1 nM estradiol (E2) is known to activate this cascade and elicit a modest, reversible potentiation of S-C fEPSPs (Woolley, 2007; Wang et al., 2016a). In vehicle-treated female mice, we replicated the E2-potentiation effect and verified it is blocked by ERα antagonist MPP (3 μM) (Fig. 6E; E2 vs E2 + MPP *p* = 0.017, Bonferroni post hoc test; veh + E2 vs veh + MPP *p* = 0.024, paired *t*-test for last 10 min of recordings). We then tested effects of E2 infusion in females given aTHC and found the enhancement of CA1 field responses was absent (Fig. 6E; *p* < 0.001, aTHC+E2 vs Veh + E2, one-way ANOVA Bonferroni post hoc) with responses reduced to the level observed with MPP infusion (Fig. 6E; *p* > 0.05 for aTHC+E2 compared to Veh + E2 + MPP, aTHC+MPP, and Veh + MPP, Bonferroni post hoc test). Infusion of MPP alone was without effect on fEPSP size in females pretreated with vehicle or THC (*p* = 0.474 and *p* = 0.073, respectively, paired t-test for means of last 10-min of recording vs mean of last 10 min of preinfusion measures). We conclude that aTHC exposure caused a lasting defect in ERα signaling that is critical for female CA1 response enhancement and LTP. Evidence that males do not rely upon this signaling for LTP, despite having high levels of hippocampal estrogen (Hojo et al., 2009; Mukai et al., 2010; Kato et al., 2013; Tabatadze et al., 2014) and moderate concentrations of synaptic ERα (Wang et al., 2018c), provides one reasonable explanation for the strikingly sexually dimorphic effects of aTHC on S-C LTP.

#### Lateral perforant path.

STP and LTP were virtually identical in the LPP of Veh- and aTHC-treated male rats (Fig. 6F). In contrast, in aTHC-treated male mice STP was severely impaired (*p* = 0.002 vs Veh) and potentiation was fully lost 3–4 min later (Veh: +42.64 ± 8.02%, THC: +3.15 ± 6.10%; *p* = 0.002) (Fig. 6G). In Veh-treated female rats and mice, LPP LTP was comparable in size to potentiation in CA1 whereas in aTHC-treated females STP was present and comparable to Veh-controls but potentiation failed to stabilize and by 60 min LTP was significantly lower than in vehicle-treated animals (mice *p* = 0.0004, rats *p* = 0.0063) (Fig. 6H,I).

### 3.5. Effects of aTHC on memory

**Fig. 7.**
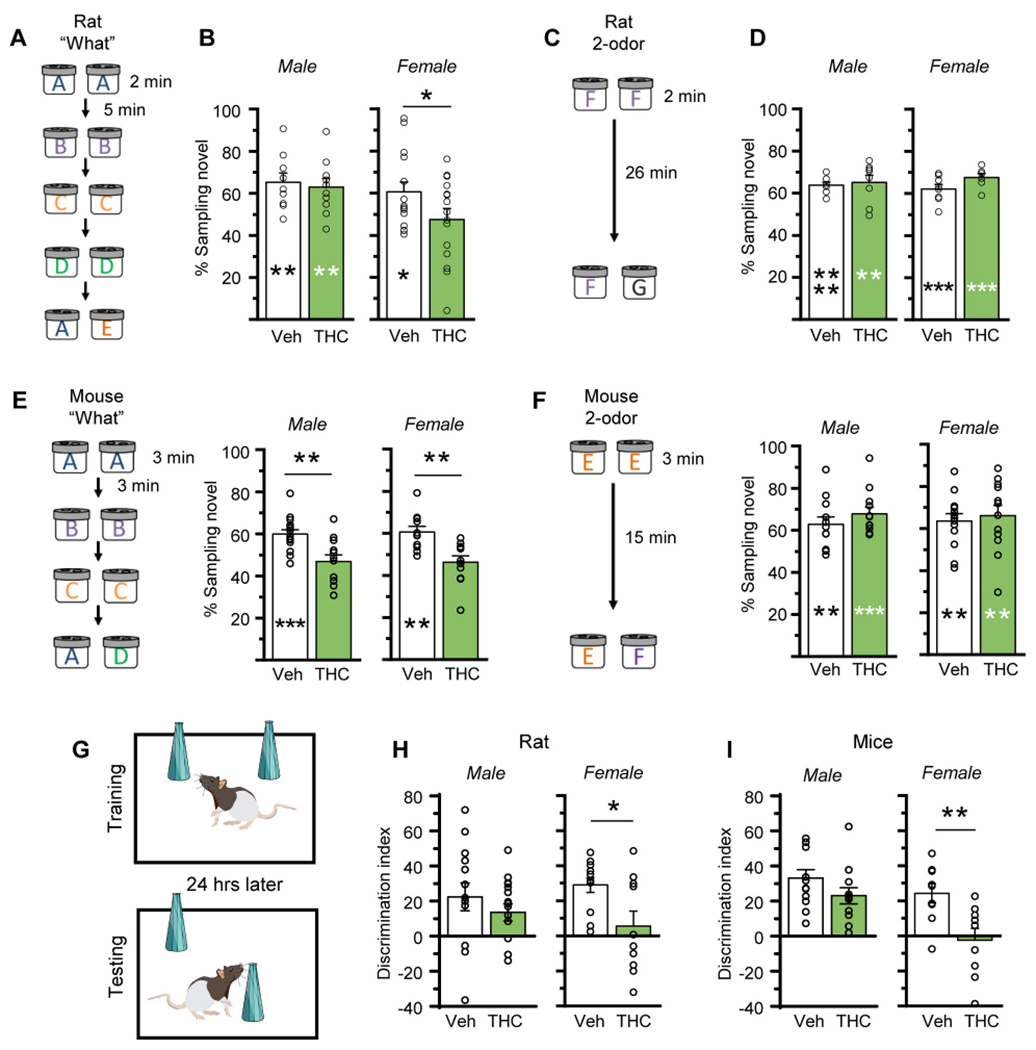
Adolescent THC treatment caused enduring impairments in episodic and spatial memoiy. (A) Schematic of the serial odor ‘what’ task for rat. (B) Vehicle (Veh) and aTHC-treated males similarly distinguished novel odor E from previously sampled odor A (Veh *n* = 9, t_8_ = 3.42, **p = 0.009 on bar for within group comparison time with novel vs familiar odor; THC *n* = 10, t_9_ = 3.13, **p = 0.006; t_17_ = 0.38, *p* = 0.71 between groups). Veh-female rats discriminated the novel cue but those receiving aTHC did not (Veh *n* = 16, t_15_ = 3.42, *p = 0.019; THC *n* = 15, t_14_ = 2.26, *p* = 0.47; *p = 0.036 between groups). (C) Rat 2-odor discrimination task. (D) In the 2-odor task, all rat groups preferentially explored the novel odor (*Male;* Veh *n* = 8, t_7_ = 9.46, ****p < 0.0001 on bar; THC *n* = 8, t_7_ = 4.45, **p = 0.003. *Female;* Veh *n* = 8, t_7_ = 5.5, ***p < 0.001; THC *n* = 7, t_5_ = 8.5, ***p < 0.001). (E) *Left*. Schematic of‘what’ task for mice. *Right*. Veh-treated male and female mice preferentially explored novel odor D vs familiar odor A whereas those receiving aTHC did not (*Male*: Veh *n* = 15, t_14_ = 4.68, ***p = 0.0004; THC *n* = 12, t_11_ = 0.98, *p* = 0.35; t_25_ = 3.57; **p = 0.0016 between groups; *Female*: Veh *n* = 11, t_10_ = 4.03, **p = 0.002; THC *n* = 11, t_10_ = 1.21, *p* = 0.30; t_29_ = 1.86, **p = 0.002 between groups). (F) *Left*. 2-odor discrimination task for mice. *Right*. All groups preferentially explored the novel odor at testing (*Male*: Veh *n* = 12, t_11_ = 3.77, **p = 0.003; THC *n* = 12, t_11_ = 5.73, ***p = 0.0001; *p* = 0.29 between groups; *Female*: Veh *n* = 13, t_12_ = 3.84, **p = 0.0024; THC *n* = 12, t_11_ = 3.25, **p = 0.008; *p* = 0.68 between groups). (G) Object Location Memory (OLM) task. (H) For male rats, OLM discrimination indexes (DIs) did not differ between Veh and aTHC groups (Veh *n* = 14, THC *n* = 15; t_20_ = 2.58, *p* = 0.33); both groups preferentially explored the moved object (Suppl. Table 1). For female rats, those that received aTHC had significantly lower DIs than Veh-controls (Veh *n* = 12, THC *n* = 10; t_20_ = 2.58, *p = 0.018) and failed to discriminate the moved object (Suppl. Table 1). (I) For male mice, Veh and aTHC groups similarly discriminated the moved object (*n* = 12/group; t_22_ = 1.51, *p* = 0.15; Suppl. Table 1) whereas for females, Veh-treated mice discriminated the moved object whereas aTHC-treated mice did not (*n* = 9/group; t_16_ = 3.06, **p = 0.007; Suppl. Table 1). For panels B, D, E, F asterisks on the bars denote within group comparisons for time sampling the novel vs familiar odor using the 2-tailed paired *t-test*, and asterisks above bars denote between group comparisons that used the 2-tailed unpaired *t-test*. Mean ± SEM values shown. See Suppl. Figs. 4 and 5 for individual animal sampling times and estrous cycle staging.

**Fig. 8.**
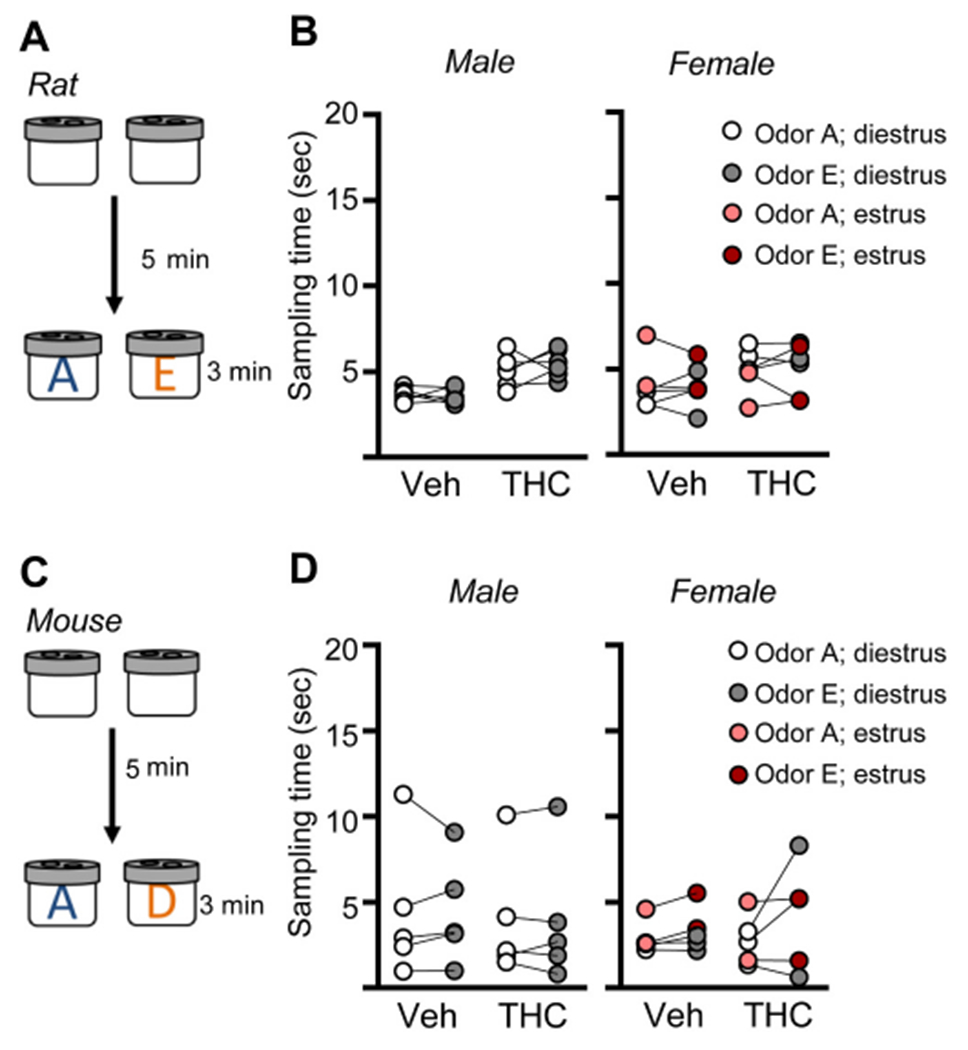
Rats and mice show no preference for individual odors used at testing in the Serial What task. (A) To assure that rats exhibited no preference for either of the two odors used at testing in the Serial What task, exploration times of odors A and E were measured for rats with only prior exposure to habituation (empty) odor cups. (B) Rats exhibited no preference for odor A or E with both males and non-proestrus females spending comparable amounts of time sampling the two odors (Male: Veh: *n* = 6, t_5_ = 0.20, *p* = 0.85; THC: *n* = 6, t_5_ = 0.89, *p* = 0.41; Female: Veh: *n* = 6, t_5_ = 0.004, *p* = 0.99; THC *n* = 6, t_5_ = 0.18, *p* = 0.87; paired t-test within groups). (C) Similarly mice with prior habituation to empty cups were tested for time exploring odors A and D, the same testing pair used in the mouse Serial What task. (D) Untrained male and female mice exhibited no preference for odor A or D (Male: Veh: *n* = 5, t_4_ = 0.02, *p* = 0.99; THC: *n* = 5, t_4_ = 0.22, *p* = 0.98; Female: Veh: *n* = 5, t_4_ = 0.74, *p* = 0.49; THC *n* = 12, t_4_ = 1.32, *p* = 0.26 paired *t-test* within groups). Individual animal measures shown; the lines link measures for the same rodent.

The hippocampus is critical for encoding episodic memories in humans (Squire, 1992; Eacott and Easton, 2010; Eichenbaum et al., 2012; Ekstrom and Ranganath, 2018) and episodic-like memory in rodents (Dere et al., 2005; Eichenbaum and Fortin, 2005; Allen et al., 2014). Thus, we tested if aTHC-related disturbances in hippocampal synaptic plasticity described above were associated with changes in acquisition of episodic memory. We have shown that the LPP is essential for acquiring cue identities (episodic ‘what’ information) in a serial-odor task in which animals sample four odor pairs in sequence (A:A; B:B; C:C; D:D) and, at testing, are presented with familiar odor ‘A’ paired with a novel odor ‘E’ (Fig. 7A). Rodents preferentially explore novel cues and accordingly spend more time with novel odor E if they remembered the originally encountered items (Cox et al., 2019; Amani et al., 2021; Quintanilla et al., 2021). Prior work has shown that, in rat, repeated adolescent cannabinoid exposure does not influence the preference for novelty (Fox et al., 2009). Additionally, studies determined that the odors used here at testing are of comparable interest to rats (Quintanilla et al., 2021) and mice (Amani et al., 2021). To assure this is the case for animals that received aTHC, groups of rats and mice (Veh- and aTHC treated) were exposed to the specific odor pairs (without prior sampling) to test for preferential exploration. In all groups, the time spent exploring the two test cues was evenly balanced (Fig. 8).

In the Serial Odor task, Veh-treated rats exhibited a strong preference for novel odor E vs familiar odor A (*p* = 0.009 for males; *p* = 0.019 for females). In males, aTHC did not disrupt this preference or alter the percent time investigating the novel cue (Fig. 7B; see Suppl. Fig. 4 for raw sampling times). In contrast, females given aTHC did not exhibit a preference for the novel odor (*p* = 0.47) and the percent time spent investigating novel odor E was higher in vehicle- vs aTHC-female rats (60.6 ± 4.7% vs 47.6 ± 5.2%, respectively; *p* = 0.036) (Fig. 7B). In a simple 2-odor discrimination task, performance of aTHC-treated rats was comparable to those pretreated with vehicle (Fig. 7C,D), indicating aTHC did not disturb odor detection, discrimination, or recall. Overall, for rats aTHC disrupted encoding the identities of serially presented cues in females only.

In the Serial ‘What’ task, Veh-treated male mice preferred the novel odor in retention testing (*p* = 0.0004), but this preference was entirely absent in the aTHC group (*p* = 0.35). The percent time exploring the novel odor was substantially greater in vehicle- than aTHC-treated male mice (*p* = 0.0016). Similarly, Veh-treated female mice preferentially explored the novel odor at testing (*p* = 0.002) but this preference was absent in aTHC-treated females (*p* = 0.30), and the percent time sampling the novel cue was greater in vehicle vs aTHC mice (60.6 ± 2.6% vs 46.3 ± 3.0%, respectively; *p* = 0.0029, unpaired *t-test*) (Fig. 7E; Suppl. Fig. 5 for raw sampling times). In the 2-odor task, Veh- and aTHC-mice, of both sexes, discriminated the novel from the familiar odor with no differences between groups (Fig. 7F).

The Object Location Memory (OLM) task was used to assess the effects of aTHC on spatial learning, a task shown to be CA1-dependent (Barrett et al., 2011; Babayan et al., 2012; McNulty et al., 2012) (Fig. 7G). The discrimination indexes of vehicle and aTHC groups were similar for male rats and mice, and both groups discriminated the novel object. In contrast, aTHC treatment impaired OLM in female rats and mice: Veh-treated females preferentially explored the novel-location object whereas aTHC-treated females did not (Fig. 7H,I; Suppl. Figs. 4 and 5 and Suppl. Table 1 for raw sampling times).

## 4. Discussion

The present studies evaluated the effects of aTHC exposure in rats and mice on increasingly complex aspects of synaptic physiology for two axonal systems in the young adult hippocampus. There was no effect of aTHC on input/output curves indicating that, for rodent S-C and LPP systems, the treatment did not disrupt basic mechanisms of synaptic transmission. There were, however, enduring aTHC effects on complex patterns of activity that normally occur during behavior and aTHC-associated impairments in two, mechanistically distinct forms of synaptic plasticity. Male rats were the least affected: aTHC did not alter frequency facilitation with trains of single pulse stimulation, theta burst responses, or LTP for either projection system (see Table 2 for summary). Male mice were anomalous: For this group alone, aTHC enhanced frequency facilitation and within theta train facilitation in field CA1 without effect on frequency facilitation in the LPP. Nevertheless, aTHC profoundly depressed LTP in the male mouse LPP. This dissociation of aTHC treatment effects on frequency facilitation and potentiation suggests that although the alterations in within-train responses may influence hippocampal circuit activity, most particularly during behaviors (retrieval, response to salient cues) that elicit beta and gamma frequency oscillations (Rangel et al., 2015; Alexander et al., 2018), these effects do not account for the marked influence of aTHC on LTP. Females of both species exhibited the most striking changes in synaptic plasticity with aTHC treatment: LTP was severely depressed in the two test pathways and this occurred without robust changes in other measures (Table 2). The results thus establish that the lasting effects of aTHC are markedly different between sexes and between rats and mice, and that daily exposure to a moderate dose of the phytocannabinoid (Huestis and Cone, 2004; Ruiz et al., 2020) during adolescence impairs mechanisms of enduring, memory-related synaptic plasticity within hippocampus without evident effects on basic synaptic transmission. This is to our knowledge the first report that aTHC impairs adult LTP (it reportedly does not affect potentiation in prefrontal cortex (Rubino et al., 2015).

Effects of aTHC on individual theta burst responses and theta train facilitation in mouse field CA1 suggest that early life THC exposure reduced GABAergic inhibition. In aTHC treated mice, the observed increase in response to later pulses in a theta burst is consistent with reduced GABA_A_R-mediated inhibition in females (Arai et al., 1995) whereas the increase in theta train facilitation suggests reduced GABA_B_R-mediated effects on release in males (Larson and Lynch, 1986; Davies et al., 1991). These results in mice accord with evidence for reduced GABAergic function, and markers of GABA transmission, in adult prefrontal cortex after aTHC treatment (Zamberletti et al., 2014; Renard et al., 2017). The absence of similar aTHC effects in rat, in the present studies, combined with reported increases in GABA release and receptor expression in rat hippocampus following adolescent CB_1_R agonist treatment (Higuera-Matas et al., 2012) suggests there may be species differences in aTHC effects on inhibition in this structure. To further test this possibility, characterization of aTHC effects on GABAergic synapses in rat and mouse hippocampus is an important target for future studies.

In aTHC-treated male mice, LPP potentiation was profoundly reduced within the first 2 min after induction and then fell to baseline values. These findings suggest that aTHC affected signaling activities during or immediately after the high frequency stimulation used to induce potentiation. For the LPP in particular, buffering postsynaptic calcium or infusion of an NMDAR antagonist, but not mGluR5 or CB_1_R antagonists, suppresses the earliest stages of potentiation in the manner seen with aTHC treatment (Wang et al., 2016b). We hypothesize that aTHC exposure altered developmental programs relating to activation of postsynaptic NMDARs in the LPP field and/or the elevation of intracellular calcium with high frequency stimulation. Why such changes would occur in LPP of male mice but not male rat or female mice is an open question, but substantial mouse vs. rat differences in the granule cell targets of the pathway have been described (Snyder et al., 2009). There is evidence that, with different treatment regimens, aTHC reduces NMDAR ligand (e.g., MK801) binding (Rubino et al., 2009; Higuera-Matas et al., 2012) but increases synaptic GluN2B levels (Zamberletti et al., 2016) in rat hippocampus but further work is needed to determine if changes in NMDAR function in the LPP field persist into adulthood and align with disturbances in STP.

The striking male-female differences in aTHC effects on potentiation of the S-C afferents to field CA1 were not entirely unexpected because potentiation in this system is sexually dimorphic, requiring local estrogen production and estrogen signaling via postsynaptic ERα in female but not male rodents (Vierk et al., 2012; Wang et al., 2018c; Gall et al., 2021). Activation of this signaling with estradiol infusion was shown here to be defective in CA1 of aTHC-treated females. This result suggests that a loss of synaptic estrogen action largely accounts for effects of aTHC exposure on S-C LTP in females and highlights the importance of identifying, in future studies, synaptic elements (receptors, signaling intermediaries) underlying impaired estrogen action. THC reportedly suppresses estrogen signaling via ERα in peripheral tissues by upregulating the competitive estrogen receptor β (ERβ) (Takeda et al., 2013). A mechanism of this kind, operating during development, could result in a lasting depression of estrogen actions required for female but not male LTP. Alternatively changes could be evident downstream from the estrogen receptors. In field CA1, estrogen infusion leads to transactivation of the neurotrophin receptor TrkB (Wang et al., 2016a) and ERα antagonism prevents synaptic TrkB activation with TBS in females only (Wang et al., 2016a). Thus impaired signaling downstream from ERα could disrupt the engagement of TrkB leading to a failure of LTP in females only.

Given the fundamental differences in mechanisms of LTP in the S-C and LPP systems it is quite likely that the molecular bases of aTHC effects also differ. The well-characterized field CA1 LTP is expressed as changes in AMPAR function in the postsynaptic element (Kauer et al., 1988; Muller and Lynch, 1988; Manabe and Nicoll, 1994; Lu et al., 1998; Granger and Nicoll, 2014). Although mechanisms and levels of field CA1 LTP are modulated by endocannabinoids (Carlson et al., 2002; Slanina et al., 2005; Monory et al., 2015) and contribute to metaplasticity in this system (Chevaleyre and Castillo, 2004), these agents and the CB_1_R are not required for induction or expression of S-C potentiation (Chevaleyre and Castillo, 2004; Slanina et al., 2005; Lynch et al., 2013; Wang et al., 2016b; Nicoll, 2017). In contrast, LPP LTP is endocannabinoid-dependent in both rats and mice and is expressed presynaptically as an increase in neurotransmitter release. Specifically, 2-arachidonoyl glycerol (2-AG) serves as the retrograde messenger to presynaptic CB_1_Rs that, in coordination with presynaptic integrins, recruit integrin-related kinases to effect an actin-dependent increase in neurotransmitter release (Wang et al., 2016a; Wang et al., 2018b). Unlike S-C potentiation, it is not known if LPP potentiation is sexually dimorphic or if, in females, it depends on estrogen function. Adolescent THC treatments do not have persistent effects on 2-AG levels (Rubino et al., 2015; Bara et al., 2021) and, although adolescent exposure causes transient and widespread reductions in the density and function of CB_1_Rs in male and female rats (Rubino et al., 2008; Rubino et al., 2015; Silva et al., 2015), both measures and CB_1_R gene expression reportedly return to normal in adult hippocampus and prefrontal cortex (Higuera-Matas et al., 2012; Rubino et al., 2015). Sustained reductions in CB_1_R levels within the LPP field would provide a reasonable explanation for impairments in female LPP LTP. Alternatively, aTHC treatment in females could shift CB_1_R signaling bias away from the integrin kinases (Wang et al., 2018b) and toward the more typical Munc18–1 target (Toonen, 2003; Schmitz et al., 2016), in essence bringing the system in line with CB_1_R activities in other sites including CA1, and thereby removing a key factor that stabilizes potentiation in the LPP.

Previous studies using the Serial Odor task employed in the present studies showed that transient silencing of the LPP completely blocks acquisition of the ‘what’ (cue identity) element of episodic memory (Cox et al., 2019) and that LPP potentiation is required for olfactory encoding in this task (Wang et al., 2018a). Consistent with these observations, we found that the striking loss of LPP LTP with aTHC exposure was accompanied by defective ‘what’ learning: groups with aTHC-associated deficits in LPP-LTP (female rats and mice, male mice) performed at chance levels in the serial odor episodic task whereas aTHC-treated male rats, that exhibited normal LPP-LTP, had ‘what’ retention scores that were equal to those of vehicle controls. Similar associations were evident for the integrity of field CA1-LTP and spatial, OLM learning: aTHC-treated male rats and mice exhibited both robust CA1-LTP and normal, field CA1-dependent (Barrett et al., 2011; Babayan et al., 2012) OLM whereas aTHC-treated female rats and mice had impaired CA1-LTP and failed OLM. The latter result is in agreement with reported effects of aTHC on spatial learning using different aTHC treatment and behavioral paradigms (Zamberletti et al., 2014). Thus, regional defects in LTP described here were predictive of learning impairments and, clearly, the electrophysiological and behavioral consequences of aTHC exposure varied across telencephalic systems.

In rodents, the loss of episodic ‘what’ encoding by LPP silencing has been shown to disrupt acquisition of other episodic elements including acquisition of spatial and temporal information (Cox et al., 2019) whereas one can selectively block ‘where’ or ‘when’ acquisition by silencing other hippocampal subsystems (Cox et al., 2019). Studies with humans have described negative, later life effects on episodic memory following prolonged cannabis use in adolescence (Fletcher and Honey, 2006; Crane et al., 2013a; Crane et al., 2013b; Riba et al., 2015) but have not isolated effects on the three basic episodic elements evaluated in rodent work. However, reported deficits in the recall of personal narratives imply that, in human, problems with proper encoding of cue identities (what) are present. The possibility of pronounced sex differences in human episodic memory after adolescent cannabis usage has not been addressed. The long-standing issue of whether rats or mice are the better model for predicting human outcomes introduces a further complexity. A survey of the literature suggests that the rat is more translationally relevant with regard to hippocampal development and behavioral flexibility (Snyder et al., 2009). If this holds true for episodic memory, then results described here would predict that early life exposure to cannabis, even at moderate doses, will have larger effects in women than men with regard to encoding the continuous flow of everyday events into organized episodes. This possibility takes on added significance given evidence that episodic memory plays a critical role in diverse aspects of high cognitive function (Crane and Goddard, 2008).

In conclusion, we have shown that aTHC exposure has persistent effects on two, mechanistically distinct forms of LTP within hippocampus that are predictive of deficits in spatial and episodic memory encoding in adulthood. Results also demonstrate that these effects are more pronounced in females than males and suggest underlying mechanisms. Together these findings provide insights as to specific risks of daily cannabis use to the adolescent brain. Goals of future research will be to identify the components of estrogen signaling that are impaired by aTHC exposure and the consequences of persistent aTHC effects on plasticity for network functions underlying complex behaviors.

## Supplementary Material

supplement

## Figures and Tables

**Table 1:** Odorants and dilutions used for behavioral studies. All odorants were diluted with mineral oil (mo) then pipetted (100 μL) onto filter paper. The scented papers were placed in odorant cups.

Odorant I.D.	Odorant (name, *company*)	Concentration (odorant: mineral oil)
A	(+) -Limonene (>97% purity, **Sigma-Aldrich*)*	1: 4000
B	Cyclohexyl ethyl acetate (>97%, *International Flavors & Fragrances Inc*.)	1.97: 4000
C	Citronellal 96% (~96%, *Alfa Aesar*)	1.5: 4000
D	Octyl aldehyde 99% (~99% *Acros Organics*)	1.5: 4000
E	Anisole 99% (~99% *Acros Organics*)	0.85: 4000
F	1-Pentanol 99% (~99% *Acros Organics*)	1.36: 4000
G	5-Methylfurfural 98% (98 + % *Acros Organics*)	2: 4000

**Table 2:** Summary of aTHC treatment effects on adult electrophysiological measures

*CA1*		Frequency Facilitation			Potentiation		Complex Responses	

Species	Sex	*10 Hz*	*20 Hz*	*40 Hz*	*STP*	*LTP*	*1 Burst*	*TBS Burst*
Mouse	Male	↑ **	↑ ****	↑ ****	-	-	-	↑*
	Female	-	-	-	-	⇓*	↑*	-

Rat	Male	-	-	-	-	-	-	-
	Female	-	-	-	⇓**	⇓****	-	⇓*
LPP		*10 Hz*	*20 Hz*	*40 Hz*	*STP*	*LTP*		
	
Mouse	Male	-	-	-	⇓**	⇓**		
	Female	-	↑*	↑*	-	⇓***		

Rat	Male	-	-	-	-	-		
	Female	-	⇓**	⇓*	-	⇓**		

The cells labeled “up” and “down” arrows indicate that aTHC significantly increased or decreased the measured response, respectively, relative to measures from vehicle-controls

* *p* < 0.05;

** *p* ≤ 0.01;

*** *p* ≤ 0.001,

**** *p* ≤ 0.0001.

Empty cells (–) indicate there was no significant effect of aTHC (p > 0.05). Results for frequency facilitation and complex responses were analyzed using RM-ANOVA (Interaction); short-term potentiation (STP) and long-term potentiation (LTP) were analyzed using the unpaired Student’s *t*-test.

## Abbreviations:

aCSF: artificial cerebrospinal fluid
aTHC: adolescent THC exposure
CB_1_R: cannabinoid receptor 1
DG: dentate gyrus
E2: estradiol
ERα: estrogen receptor α
ERß: estrogen receptor β
fEPSP: field excitatory postsynaptic potential
FF: frequency facilitation
L-E: Long-Evans
LPP: lateral perforant path
LTP: long-term potentiation
NMDAR: NMDA receptor
OLM: Object Location Memory
P: postnatal day
S-C: Schaffer-commissural
STP: short-term potentiation
THC: Δ^9^-tetrahydrocannabinol
TBS: theta burst stimulation
Veh: vehicle
