# Supplementary material for: Persistent sexually dimorphic effects of adolescent THC exposure on hippocampal synaptic plasticity and episodic memory in rodents
Source: Neurobiol Dis. Author manuscript; Available in PMC 2023 Jun 8. (PMC10249649; doi:10.1016/j.nbd.2021.105565)
Supplement: supplement [file NIHMS1903601-supplement-supplement.docx]

***Supplemental Information: Le et al.***


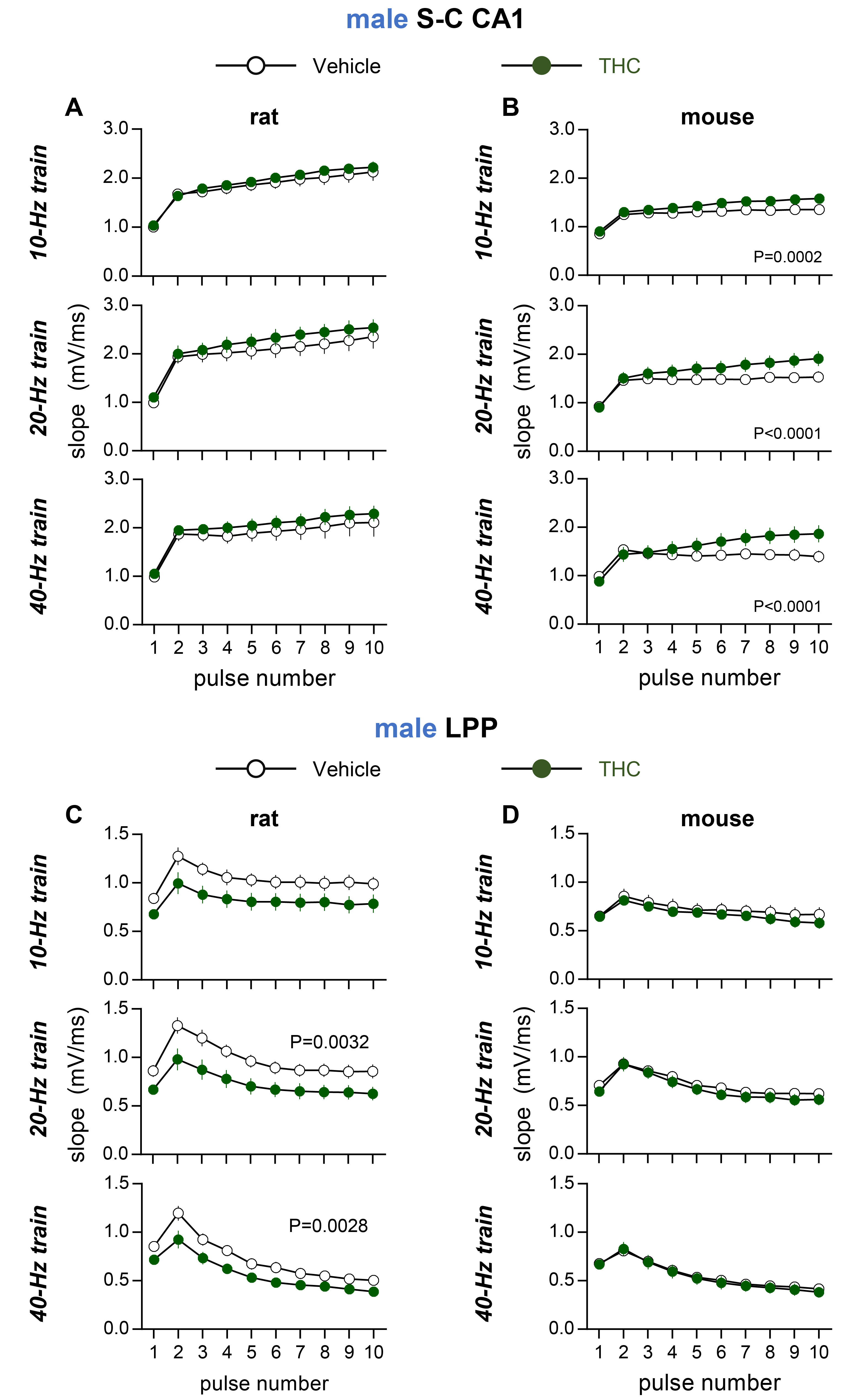


**Supplemental Figure 1. Adolescent THC exposure effects on frequency facilitation in male rats and mice: Raw values for data presented in text Figures 3-4.** (**A, B**) Field CA1 responses (fEPSP slope) to 10 pulse Schaffer-commissural (S-C) stimulation at 10, 20, and 40 Hz all show frequency facilitation. (**A**) Plot of raw values shows S-C frequency facilitation is comparable in THC-treated and vehicle-treated male rats (10 Hz: F_9,135_=0.48 P = 0.89; 20 Hz: F_9,135_=0.38 P=0.94, 40 Hz: F_9,126_= 0.11 P=0.99) (Vehicle N=10, THC N=7). (**B**) S-C frequency facilitation is slightly larger in THC- as compared to vehicle-treated male mice (10 Hz: F_9,108_=4.03 P=0.0002; 20 Hz: F_9,108_=5.92 P<0.0001; 40 Hz: F_9,108_=10.02 P<0.0001) (Vehicle N=7, THC N=7). (**C, D**) Stimulation trains were applied to the lateral perforant path (LPP) and field EPSP responses were recorded from the dentate gyrus outer molecular layer. (**C**) With stimulation at 20 and 40 Hz, frequency facilitation was smaller in slices from THC-treated as compared in vehicle-treated male rats (10 Hz: F_9,117_=1.26 P=0.27, 20 Hz: F_9,117_= 3.02 P=0.0032, 40 Hz: F_9,117_= 2.97 P=0.0028) (Vehicle N=7, THC N=8). The effect is not significant when normalized to the first value (see Figure 3C) suggesting that differences in the first fEPSP slope give rise to the group effect. (**D**) LPP frequency facilitation did not differ between THC-treated and vehicle-treated male mice (10 Hz: F_9,117_=1.46 P=0.17; 20 Hz: F_9,108_=0.95 P=0.49; 40 Hz: F_9,117_=0.27 P=0.98) (Vehicle N=8, THC N=7). The 2-way ANOVA was used for statistical analysis with significance for Interaction presented. N represents the number of slices per group; group mean ± SEM values presented.


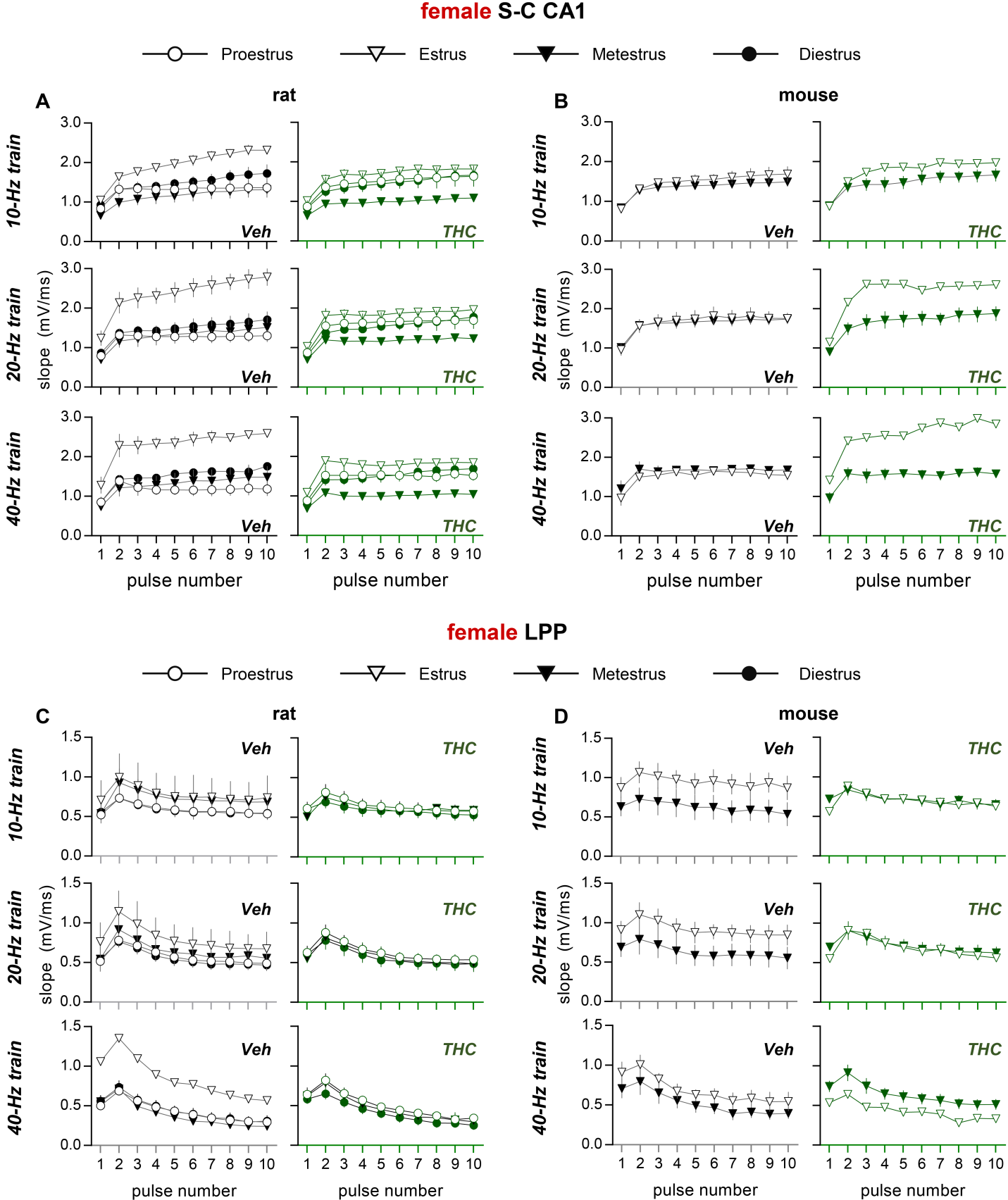


**Supplemental Figure 2. Adolescent THC exposure effects on frequency facilitation in female rats and mice: Raw values and estrous stage breakdown for data presented in text Figures 3C, D and Figures 4C, D.** (**A, B**) Field CA1 responses (fEPSP slope) to 10 pulse Schaffer-commissural (S-C) stimulation at 10, 20, and 40 Hz show frequency facilitation. (**A**) Raw values for the pooled data presented in text Figure 3C show responses in female rats given vehicle (left) or THC (right, green). The number of slices (N) evaluated are as follows (for veh, THC): proestrus N=6,3; estrus N=2,4; metestrus N=3,3; diestrus N=3,4. (**B**) Raw values and estrous cycle breakdown for the data presented in text Figure 4C showing S-C responses in female mice: estrus N=4,1; metestrus N=3,6. (**C,D**) Dentate gyrus outer molecular layer responses (fEPSP slope) to 10 pulse stimulation of the LPP at 10, 20, and 40 Hz show initial frequency facilitation followed by a decline in response size that is most evident at higher frequencies. (**C**) Raw values for female rat results presented in text Figure 3D (Numbers of slices per group (N): proestrus N=6,8; estrus N=2,0; metestrus N=2,2; diestrus N=6,6). (**D**) Raw values for female mouse results presented in text Figure 4D (estrus N=3,1; metestrus N=3,5). The N values represent number of slices per group; group mean ± SEM values shown.


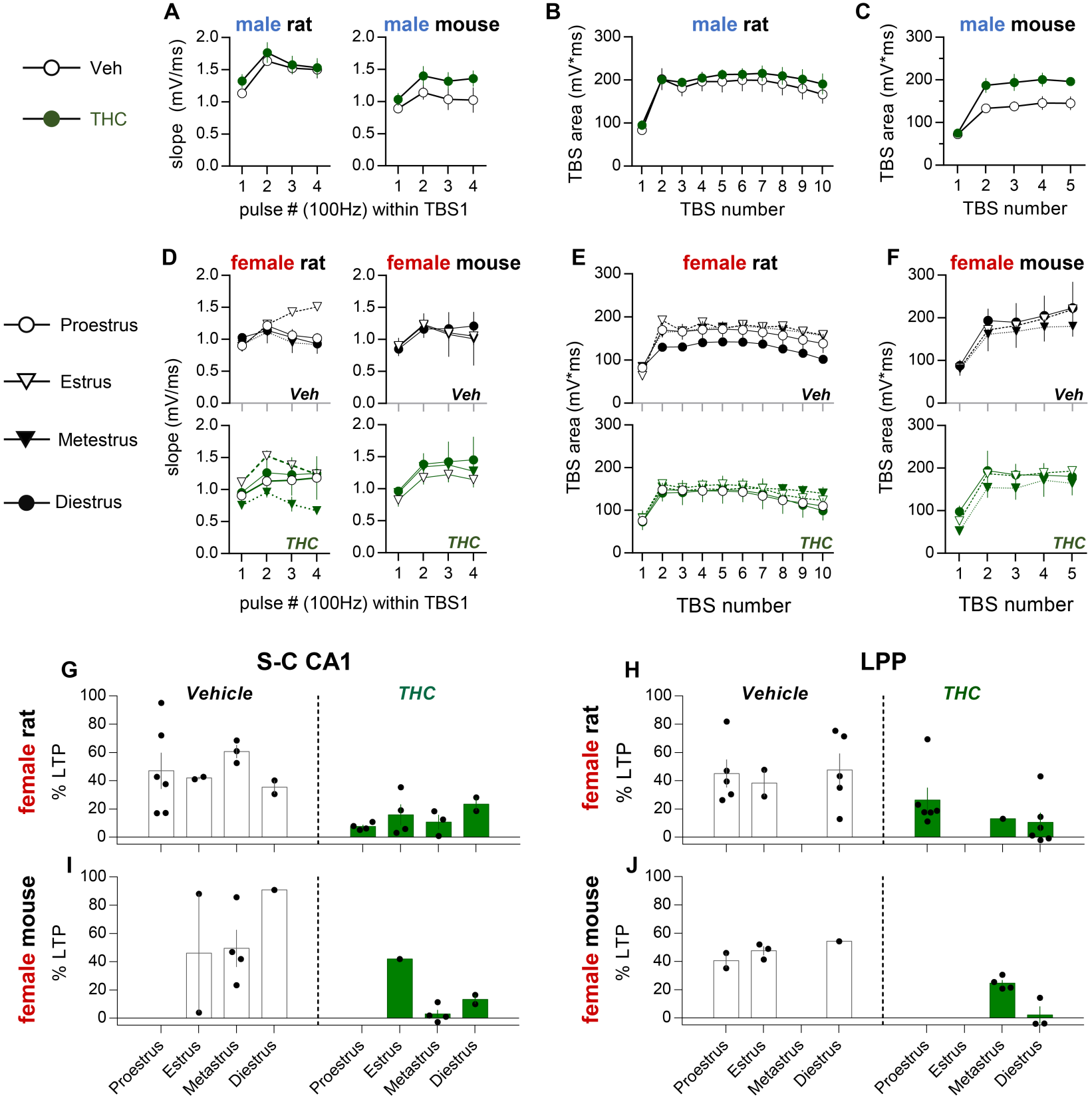


**Supplemental Figure 3. THC treatment effects on theta burst responses: Graphs show raw values for the size of theta burst responses and the breakdown of estrous cycle stage for female cases (that are shown pooled by treatment group in text Figures 5 and 6).** (**A-G, I**) Male and female rodents were given either THC or vehicle during adolescence and then tested for LTP of the Schaffer-collateral (S-C) CA1 projection using theta burst stimulation (TBS) (i.e., ten bursts delivered at 5 Hz with each burst consisting of four pulses at 100 Hz). (**A**) THC treatment did not influence the size (fEPSP slope) of CA1 responses to individual pulses within the first burst (2-way ANOVA Interaction: rat F_3,36_=0.67 P=0.58; mouse F_3,30_=0.92 P=0.45) (rat N=7/group, mouse N=6/group). (**B,C**) Measures of the area of individual burst responses during the 10 burst train show that adolescent THC treatment did not influence the overall response size in male rats (B; F_9,108_=0.37 P=0.95) but did enhance within-train facilitation of burst responses in male mice (C; F_4,40_=5.25 P=0.0017). (**D-F**) Plots show raw values for the size of single burst responses for the first burst (**D**) and for a 10 burst train (**E,F**) for females with values plotted by estrous stage (these show estrous breakdown for pooled data presented in text figures 5B-5F); E and F show within-train frequency facilitation in all groups. The N values for data in D-F (vehicle, THC) are as follows. Rat: proestrus N=5,4; estrus N=1,4; metestrus N=3,3; diestrus N=2,2. Mouse: estrus N=21; metestrus N=4,4; diestrus N=1,2. The groups were not powered to determine the significance of estrous state among these low circulating estrogen stages (estrus, metestrus, diestrus), thus statistical comparisons of the stages were not performed. (**G-J**) A 10 burst train of TBS (for S-C) or HFS (for LPP) was used to induce LTP in female rodent slices; bar graphs summarize the mean ± SEM fEPSP slope for single pulse responses recorded from 55-60 minutes after induction. Plots show raw values with estrous cycle breakdown for female LTP data presented in text Figure 6C (G), text Figure 6H (H), text Figure 6D (I) and text Figure 6I (J). Group mean ± SEM values shown; the N represents the number of slices evaluated per group.


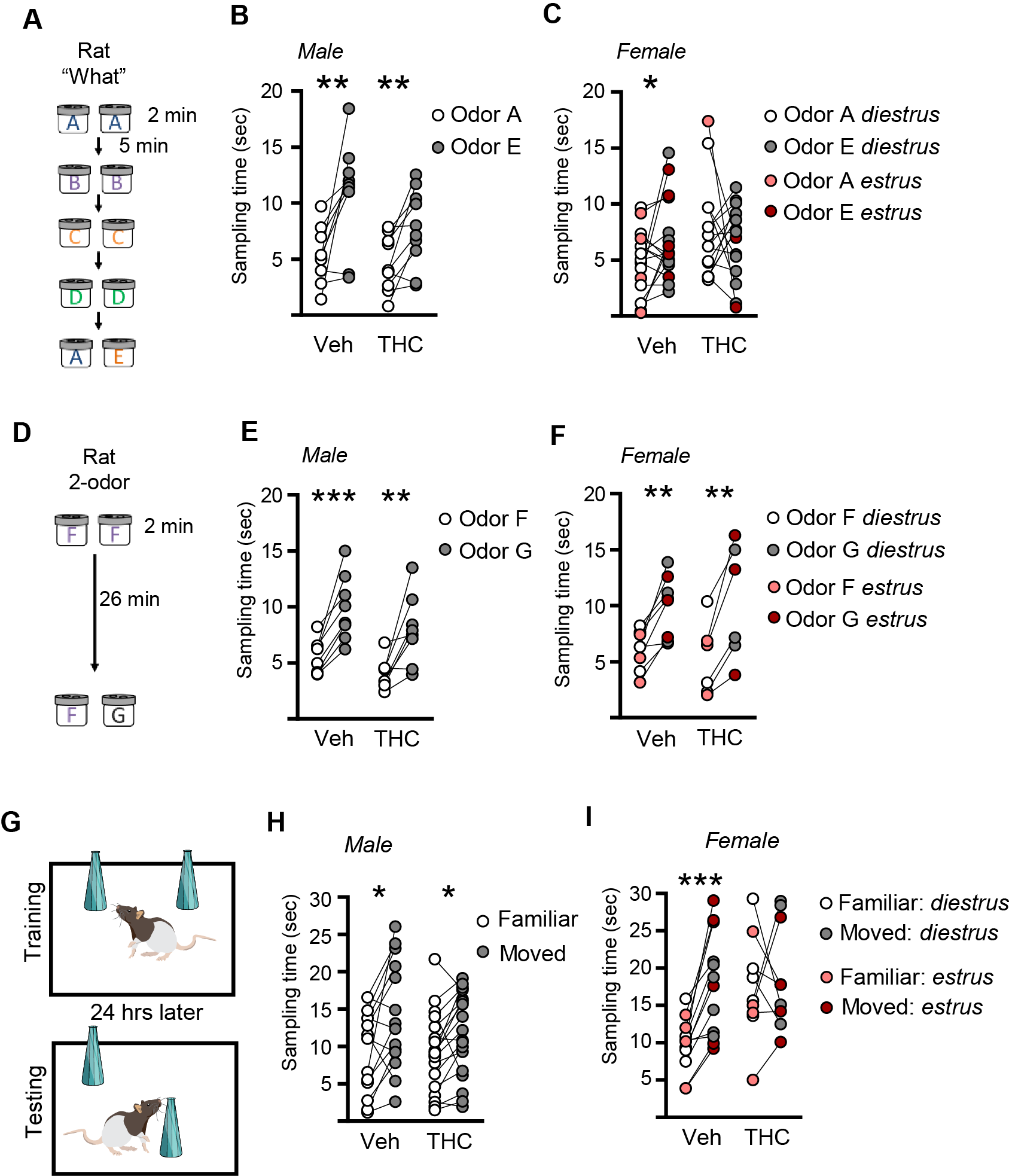


**Supplemental Figure 4. Raw sampling time measures for rat testing of ‘What’ and ‘Where’ components of episodic memory**. (**A**) The Serial ‘What’ behavioral paradigm entailed presentation of a sequence of four odor pairs (A:A - D:D) followed by a testing pair (A:E). (**B**) Raw values for male rat testing on the serial ‘What’ task shown preferential exploration of the novel odor E compared to the familiar odor A in vehicle- and THC-treated groups thereby denoting learning by both (Veh: n= 9, t_8_= 3.43, **p=0.009; THC: n=10, t_9_=3.59, **p=0.006). In the plot, lines link the times sampling familiar odor A and novel odor E for the same animal. (**C**) Vehicle-treated female rats preferentially sampled novel odor E compared to familiar odor A whether in estrus or diestrus, whereas THC-treated females demonstrated no preference between the two odors whether in diestrus or estrus (Veh: n= 16, t_15_= 2.14, *p=0.04; THC: n=15, t_14_=0.50, p=0.62). (**D**) 2-odor discrimination paradigm schematic. (**E-F**) In the 2-odor discrimination, all rat groups, regardless of treatment and sex (and for females estrous stage) spent more time exploring novel odor G than familiar odor F (Male: Veh: n=8 , t_7_=5.75 , ***p<0.001 ; THC n=8 , t_7_=3.56 , **p<0.01; Female: Veh: n=8 , t_7_=5.04 , **p=0.001 ; THC n=6 , t_5_=4.76 , **p=0.005). (**G**) Object Location Memory (OLM) task schematic. (**H**) In the OLM task, vehicle- and THC-treated male rats spent more time interacting with a novel-location (Moved) object than the familiar location object (Male: Veh: n=14 , t_13_=2.73, *p=0.017 ; THC n=18 , t_17_=2.50 , *p=0.023; paired t-test). (**I**) In the OLM task, vehicle-treated female rats spent more time exploring the moved object compared to the familiar-location object at testing regardless of estrous stage whereas females exposed to THC during adolescence did not preferentially explore the moved object at testing (Female: Veh: n=12, t_11_=5.37, ***p=0.002; THC n=10, t_9_=0.529, p=0.61; paired t-test within each group). In all panels, individual animal values are shown.


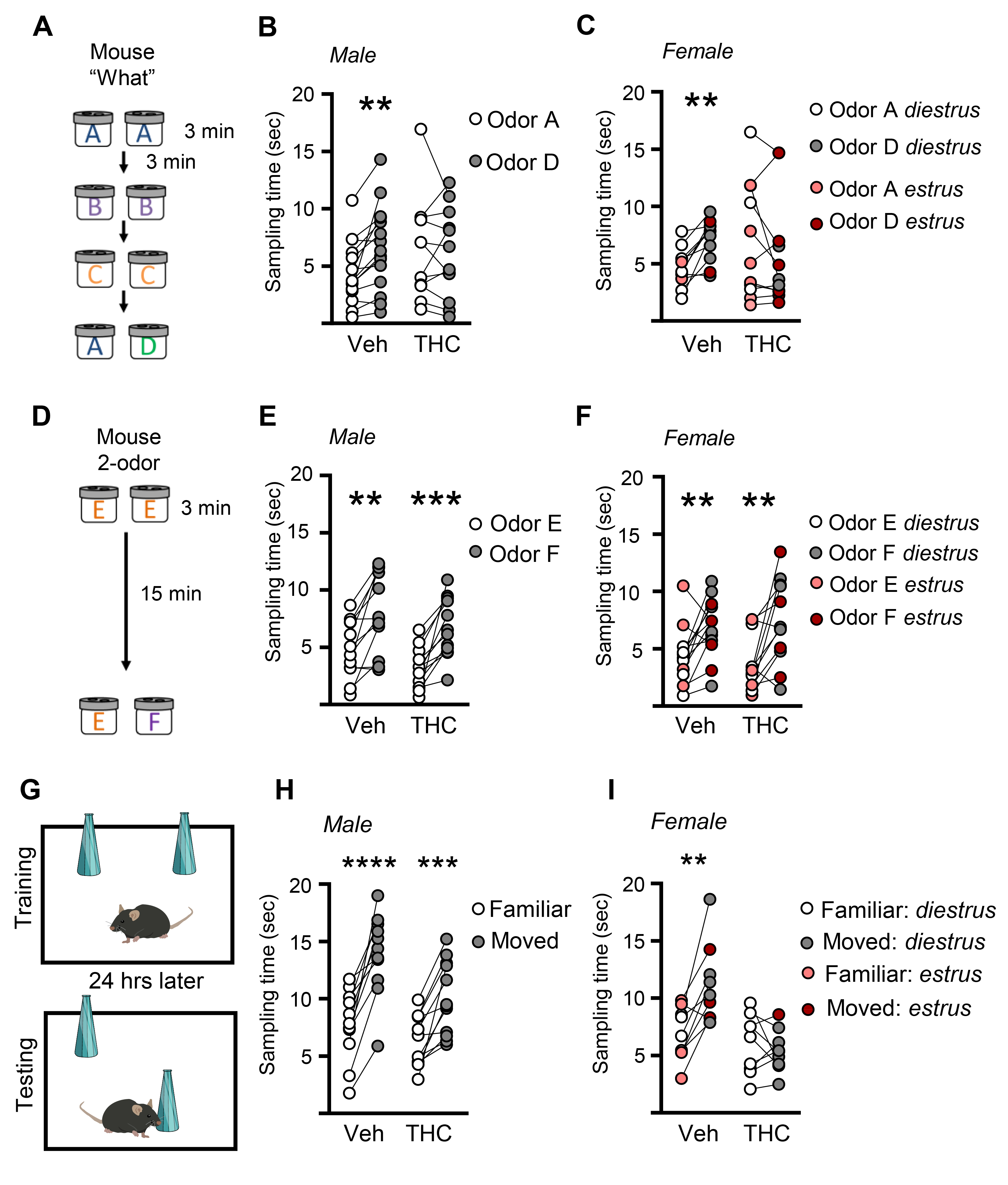


**Supplemental Figure 5. Raw sampling time measures for mouse testing the encoding of ‘What’ and ‘Where’ components of episodic memory**. (**A**) The Serial ‘What’ behavioral paradigm used a sequence of three odor pairs (A:A-C:C) followed by a testing pair containing familiar odor A and novel odor D. (**B**) When tested on the serial ‘What’ task, vehicle-treated male mice preferentially explored novel odor D compared to the familiar odor A; in contrast, THC-treated male mice spent the same amount of time with each odor (Veh: n= 15, t_14_= 3.83, **p=0.001; THC: n=12, t_11_=0.38, p=0.72; paired t-test within groups). (**C**) Raw sampling times recorded for vehicle-treated females preferentially sampled novel odor D compared to familiar odor A whereas THC-treated females showed no discrimination between the two odors (Veh: n= 11, t_10_= 4.51, **p=0.011; THC: n=11, t_10_=1.24, p=0.24). Female mice in either diestrus (gray) or estrus (red) were pooled together and animals in proestrus were dropped from analysis. (**D**) Schematic of the mouse 2-odor task conducted to ensure simple discrimination was present in both groups. (**E-F**) In the 2-odor task, all mouse groups, regardless of treatment and sex spent more time with a novel odor F than familiar E (Male: Veh: n=12, t_11_=4.25, **p=0.001 ; THC: n=12, t_11_=4.93, ***p<0.001; Female: Veh: n=13 , t_12_=3.13, **p=0.009 ; THC n=12 , t_5_=3.40 , **p=0.006; paired t-test within groups). (**G**) Testing on the Object Location Memory (OLM) task was conducted 24 hrs after training (Males 5 min training, females 10min training; near threshold for each sex). (**H**) In the OLM task, both vehicle- and THC-treated males preferentially explored the moved object (Male: Veh: n=12, t_11_=6.24, ****p<0.0001; THC n=12, t_11_=4.94, ***p=0.0004; paired t-test within groups). (**I**) In the OLM task, vehicle treated female mice also spent more time with the move object than the familiar location object whereas those treated with THC exhibited no difference in their interactions with the objects (Female: Veh: n=9, t_8_=4.10, **p=0.003 ; THC n=9, t_8_=0.71, p=0.49; paired t-test within each group). In all panels, individual animal values are shown.

***Supplemental Table 1.* Relative times spent exploring the displaced object in the object location memory (OLM) task indicates that females given THC during adolescence did not learn object location.**

| **Rat** | | **N** | **% displaced object sampling** | **Statistics (paired t-test vs % stationary)** | |  |
| --- | --- | --- | --- | --- | --- | --- |
|  | ***Male*** | |  | | |  |
| Vehicle | | 14 | 61.2% * | | t_13_= 2.85, *p=0.014 |  |
| THC | | 16 | 56.8% ***** | | t_17_=2.80, *p=0.012 |  |
|  | ***Female*** | |  | | |  |
| Vehicle | | 12 | 64.5% ******* | | t_11_=7.0, ***p<0.0001 |  |
| THC | | 10 | 52.8% n.s. | | t_9_=0.66, n.s. p=0.527 |  |

| **Mouse** | | **N** | **% displaced object sampling** | **Statistics (paired *t-test* vs % stationary)** |  |
| --- | --- | --- | --- | --- | --- |
|  | ***Male*** | |  | |  |
| Vehicle | | 12 | 66.6% ******** | t_11_=6.8, ****p<0.0001 | |
| THC | | 12 | 61.6% ******* | t_11_=5.01, ***p<0.001 |  |
|  | ***Female*** | |  | |  |
| Vehicle | | 9 | 62.2% ****** | t_8_=4.4, **p=0.002 |  |
| THC | | 9 | 48.8% n.s. | t_8_=0.36, n.s. p=0.726 |  |

The percent (%) total exploration time spent attending to the displaced object was compared within group (species, sex) to the % exploration time spent attending to the non-moved (stationary) object. Among the rat groups, male rats regardless of treatment and female rats treated with vehicle preferentially explored the moved vs the unmoved object thus indicating that the memory for object location was retained. In contrast, THC-treated female rats did not discriminate between the two objects. Similar results were obtained with the mouse groups: all male mice and vehicle-treated female mice preferentially explored the moved object whereas female mice treated with THC during adolescence did not discriminate between the two objects. Statistics were performed with the paired *t-test*.
